# Genotype × Nutritional Environment Interactions in Grazing Beef Cow-Calf Systems: Evidence for Crossbreeding and Reproductive Efficiency from Breeding to Weaning

**DOI:** 10.3390/ani16213344

**Published:** 2026-10-23

**Authors:** Morgan Radtke, Bailey Bailey, Priunka Bhowmik, Ramanathan Kasimanickam

**Affiliations:** 1 College of Veterinary Medicine, Washington State University, Pullman, WA 99164, USA; morgan.h.radtke@wsu.edu (M.R.); bailey.bailey@wsu.edu (B.B.); 2 College of Agricultural, Human, and Natural Resource Sciences, Washington State University, Pullman, WA 99163, USA; priunka.bhowmik@wsu.edu

**Keywords:** crossbreeding, heterosis, herbage allowance, nutritional robustness, reproductive efficiency, genotype × nutritional environment

## Abstract

Reproductive efficiency determines the biological and economic sustainability of grazing beef cow–calf systems, yet the extent to which nutritional conditions modify genotype-associated reproductive performance remains unclear. This review critically examines the relationships among crossbreeding, heterosis, herbage allowance, and reproductive outcomes across the breeding-to-weaning continuum. A structured narrative synthesis evaluated 36 publications, classified as direct reproductive genotype × nutritional environment (G × N) evidence (Level I, *n* = 2), genotype and crossbreeding evidence (Level II, *n* = 14), nutritional evidence (Level III, *n* = 9), and mechanistic or supportive evidence (Level IV, *n* = 11). Across the reviewed studies, crossbreeding was frequently associated with favorable reproductive and maternal outcomes, although the magnitude and consistency of these associations varied among breed combinations, production environments, and reproductive endpoints. Greater forage availability and more favorable nutritional status were also associated with improved body condition and reproductive outcomes in some studies, consistent with established metabolic and endocrine mechanisms. However, these genotype- and nutrition-associated patterns do not, by themselves, establish causal effects or G × N interactions. Only two publications directly evaluated reproductive G × N within an analytical framework, both originating from a shared grazing research program. One reported stage-specific interaction evidence, particularly during the calving-to-weaning period, while the other included an interaction term without separately reporting its estimate. Nutritional effects varied with physiological stage, parity, body condition, and previous nutritional exposure. These findings emphasize that pregnancy rate alone incompletely represents reproductive efficiency because subsequent reproductive and calf losses influence cumulative productivity. Calves weaned per cow exposed, supplemented by calf weight weaned, provides a more comprehensive endpoint. Independent, longitudinal factorial studies across diverse genotypes and grazing environments are needed to establish the reproducibility, biological significance, and management implications of reproductive G × N interactions.

## Simple Summary

Improving reproductive efficiency is essential for sustainable beef cattle production, particularly in grazing systems where forage availability varies across seasons and years. This review examines how cattle genetics, crossbreeding, and nutritional conditions influence reproductive performance from breeding through calf weaning. Crossbreeding has been associated with favorable reproductive and maternal outcomes in some populations, while nutritional status has been associated with body condition and reproductive function; the strength and consistency of these relationships vary among studies. However, favorable genetic and nutritional effects do not necessarily demonstrate that different genotypes respond differently to nutritional challenges. Evidence directly evaluating these interactions remains limited, with the strongest studies originating from a shared research program rather than independent experiments. Reproductive responses may also differ across pregnancy establishment, calving, and weaning. Therefore, calves weaned per cow exposed to breeding provides a more comprehensive measure of reproductive efficiency than pregnancy rate alone. Further research across diverse breeds and grazing environments is needed to identify nutritional robustness and improve management decisions.

## 1. Introduction

Reproductive efficiency is a major determinant of biological productivity and economic performance in grazing cattle systems [1,2,3,4]. In beef cow–calf enterprises, reproductive losses can occur at successive stages from breeding through weaning, including failure to conceive, embryonic or fetal loss, failure to calve, and preweaning calf mortality [2,5]. Consequently, pregnancy rate alone provides an incomplete measure of reproductive efficiency. Calves weaned per cow exposed to breeding provides a more integrative outcome because it incorporates losses occurring across the breeding-to-pregnancy, pregnancy-to-calving, and calving-to-weaning continuum.

Nutrition is among the most important environmental determinants of reproductive performance in grazing cattle. Unlike confined systems, grazing systems depend on forage resources that vary seasonally and year to year in quantity and quality [6,7,8]. Insufficient nutrient intake can alter energy balance and body reserves, delay postpartum ovarian activity, impair oocyte and embryo competence, compromise pregnancy maintenance, and subsequently affect lactation, maternal performance, and calf growth or survival [9,10,11,12,13]. Herbage allowance (HA), which relates available forage to animal demand, provides a useful management framework for characterizing this nutritional environment because it influences forage intake, dietary selection, energy balance, and body-condition trajectories [14,15,16].

Genetic strategies can also improve reproductive and maternal performance. Crossbreeding exploits heterosis and breed complementarity and can be particularly valuable for low-heritability fitness traits such as fertility, survival, and longevity [17,18,19,20]. Crossbred females have frequently demonstrated favorable reproductive and maternal performance relative to purebred contemporaries, although responses vary with breed composition, crossbreeding system, production environment, and outcome evaluated [18,20,21,22,23]. Importantly, these advantages may arise through individual heterosis, maternal heterosis, breed complementarity, or combinations of these effects and therefore should not be considered a uniform property of all crossbred cattle.

Although the independent effects of nutrition and crossbreeding are well established, considerably less is known about whether nutritional environment modifies genotype-associated reproductive performance [24,25,26,27,28]. This distinction is fundamental. Superior performance of crossbred cattle and improved reproduction under greater nutritional availability represent genotype and nutritional main effects, respectively; they do not demonstrate a genotype × nutritional environment (G × N) interaction unless the magnitude or direction of genotype-associated performance differs among nutritional environments. Such interactions are biologically plausible because differences among genotypes in mature size, nutrient requirements, body-reserve utilization, lactational demand, maternal performance, and heterosis may influence their responses to nutritional restriction.

The potential expression of G × N may also differ across reproductive stages. Nutritional constraint around breeding may influence postpartum reproductive recovery, conception, and early pregnancy establishment, whereas restriction during gestation may affect pregnancy maintenance and fetal development. During the calving-to-weaning period, maternal nutrient requirements, lactation, body-reserve mobilization, maternal behavior, and calf survival become increasingly important. Thus, genotype-associated differences that are modest at pregnancy diagnosis may become more evident when reproductive and maternal losses accumulate through weaning. Evaluating calves and kilograms of calf weaned per cow exposed may therefore reveal biologically and economically important genotype-associated responses that are not apparent from pregnancy rate alone.

Evaluation of reproductive performance across multiple production cycles and over the productive lifetime of the cow has a longstanding foundation in beef-cattle research. Classical studies have quantified genetic and crossbreeding effects on longevity, age-specific reproductive performance, lifetime calf production, and cumulative productivity [29,30], while calves or kilograms of calf weaned per cow exposed have long served as integrative measures of cow–calf performance [31,32]. Likewise, genotype × environment studies have evaluated reproductive and maternal performance of cattle across contrasting production environments [33,34,35].

This question is increasingly relevant in grazing systems exposed to seasonal and interannual variation in forage availability and climatic conditions. Greater robustness across nutritional environments, expressed as a smaller decline in reproductive or maternal performance as nutritional conditions deteriorate, may contribute to more stable cow–calf productivity. However, robustness should be distinguished from resilience, which requires evidence of response to and recovery following a disturbance, and from adaptability to a particular production environment. Moreover, favorable performance of crossbred cattle under challenging conditions does not itself establish G × N unless differential responses among nutritional environments are demonstrated.

The primary evidence considered in this review is derived from grazing beef cow–calf systems. Selected evidence from seasonal pasture-based dairy systems is included only where it provides comparative or mechanistic insight into heterosis, nutritional status, body condition, and reproductive performance under forage-dependent conditions. Dairy reproductive endpoints are not considered directly equivalent to cumulative beef cow–calf outcomes.

Accordingly, this structured narrative evidence synthesis integrates experimental, observational, longitudinal, mechanistic, and modeling evidence to evaluate reproductive efficiency from three interconnected perspectives: (1) stage-specific reproductive attrition from breeding through weaning; (2) genotype × nutritional environment relationships, with particular emphasis on crossbreeding, heterosis, and HA; and (3) cumulative cow–calf efficiency, expressed principally as calves weaned per cow exposed. Particular attention is given to distinguishing direct evidence of G × N from evidence supporting independent genetic or nutritional effects and from mechanistic or modeling evidence. The central objective is to determine whether, when, and under which production conditions nutritional environment modifies genotype-associated reproductive performance, and how such stage-specific responses accumulate to influence cumulative cow–calf efficiency.

## 2. Literature Search and Evidence Synthesis

### 2.1. Review Design and Scope

This article presents a structured narrative synthesis of evidence informed by systematic literature-search methods. It synthesizes evidence on crossbreeding, nutritional environment, and reproductive performance in grazing cattle, with particular emphasis on genotype × nutritional environment (G × N) interactions. Experimental, observational, longitudinal, modeling, mechanistic, and review evidence was considered, with greatest emphasis on studies evaluating reproductive outcomes under pasture-, rangeland-, or forage-based conditions [36,37]. Search reporting was strengthened using PRISMA-S principles where applicable; the review was not prospectively registered or conducted as a fully PRISMA-based systematic review. No dedicated narrative-review reporting checklist was prospectively adopted; PRISMA-S principles were used selectively to improve transparency of the literature-search description.

Beef cow–calf systems constituted the primary evidence base because calves weaned per cow exposed to breeding provides an integrative measure of reproductive and maternal performance [2,3,4,5]. Selected pasture-based dairy studies were included as comparative or supportive evidence when they provided relevant information on heterosis, nutritional status, body condition, fertility, or G × N relationships [23]. Dairy reproductive outcomes were not considered directly equivalent to cumulative beef cow–calf outcomes.

The geographic scope was not restricted a priori. Relevant studies represented grazing environments in South America, North America, Europe, and New Zealand in the classified inventory; Australian work is discussed as external context, encompassing native grasslands, semi-arid and subtropical rangelands, temperate grazing systems, and intensively managed seasonal pastures [6,38,39,40,41,42].

### 2.2. Literature Search

Literature searches were conducted in PubMed, Web of Science Core Collection, Scopus, and CAB Abstracts from database inception through 16 September 2026. Searches addressed genotype and crossbreeding, nutritional environment, reproductive outcomes, genotype × nutrition/environment interactions, reproductive mechanisms, and lifetime productivity. Keywords encompassed beef cattle, breed, genotype, crossbreeding, heterosis, nutrition, grazing, herbage allowance, stocking rate, supplementation, body condition, fertility, pregnancy, calf survival, weaning, and lifetime productivity. Boolean combinations were adapted to each database, and reference lists of relevant studies and reviews were screened.

Priority was given to studies addressing reproductive performance in grazing beef cattle, genotype × nutrition interactions, crossbreeding, heterosis, and nutritional effects. Supplementary Table S1 presents nine representative search combinations, aggregate retrieval counts, and search limitations. Exact executed queries for mechanistic outcomes, lifetime productivity, and calves weaned per cow exposed were not archived; these topics were additionally explored through reference checking and narrative citation discovery. Aggregate returns represent overlapping searches conducted before screening and deduplication and cannot establish domain-specific contributions or unique record counts.

The final synthesis included 36 publication-level records documented in Data File S1, classified using the predefined Level I–IV evidence framework. Companion publications from shared experiments or cohorts were linked to avoid treating them as independent evidence. As this was a structured narrative review rather than a prospectively registered systematic review, protocol registration, duplicate independent screening, and formal risk-of-bias assessment were not performed. Historical deduplication, screening, eligibility, and exclusion counts were not retained and cannot be reliably reconstructed. Accordingly, Supplementary Figure S1 presents an evidence-identification transparency diagram, rather than a fully compliant PRISMA 2020 flow diagram.

### 2.3. Eligibility and Study Selection

Studies were considered relevant when they addressed one or more of the following in cattle: (1) genotype, crossbreeding, or heterosis in relation to reproductive or maternal performance [18,20,21,22,23,27]; (2) HA, forage availability, stocking rate, supplementation, body condition, or nutritional status in relation to reproduction [6,9,10,11,12,13,14,15,16]; (3) G × N or genotype × environment interactions [24,25]; or (4) biological mechanisms linking nutrition or heterosis with reproductive outcomes. For inventory classification, a record was included if its study-specific results were directly analyzed in the synthesis, including primary reproductive or nutrition studies and selected mechanistic, modeling, or review sources used to interpret specific findings. Citations used only for introductory background, definitions, or methods were not automatically entered into the inventory. Consequently, the evidence inventory is a purposively assembled set of discussed publications, not a representative sample of all search returns. Broader genotype × environment references, including Jeyaruban et al. (2009) [43] and other studies [33,34,35], are contextual citations rather than direct reproductive G × N records; reviews [5,24,27] likewise supply background and are not counted as independent primary evidence. This boundary is judgment-based and may omit relevant publications.

Priority was given to grazing, pasture-based, rangeland, and forage-dependent systems. Studies outside these systems were retained when they provided important mechanistic evidence concerning energy balance, body condition, metabolic–endocrine regulation, ovarian function, oocyte or embryo competence, pregnancy maintenance, maternal performance, or calf survival. Relevant outcomes included postpartum reproductive recovery, conception and pregnancy, pregnancy loss, calving, calf survival, weaning, longevity, and cumulative outcomes such as calves or kilograms of calf weaned per cow exposed [2,5].

Peer-reviewed primary studies formed the primary biological evidence base. The publication-level evidence inventory comprised the 36 publication-level records documented in Data File S1. Publications arising from the same experiment, cohort, or research program were linked where identifiable and were not interpreted as independent replication. Sources cited solely for background, methodology, mechanistic context, or broader interpretation were not treated as independent evidence units unless they were included in Data File S1. Reviews, meta-analyses, modeling studies, conference proceedings, extension publications, and technical reports were used primarily for mechanistic, conceptual, methodological, or management context [5,24,27]. Studies unrelated to cattle reproduction or biologically relevant intermediate pathways were excluded from the evidence synthesis, and studies focused exclusively on growth or carcass traits were considered only when they informed heterosis, breed complementarity, feed efficiency, or adaptation [17,25,26,27,28].

#### Evidence-Inventory Transparency and PRISMA-S-Informed Reporting

The final evidence inventory comprised 36 publication-level records selected for relevance to genotype, nutritional environment, reproductive performance, or associated biological mechanisms in grazing cattle. Each publication was retained as a separate record and assigned to one of four evidence levels according to the endpoints evaluated and the directness of its interaction analysis to the review question. To assess nonindependence, publications were compared by experimental location, study period, animal population, treatment allocation, and outcomes; related records were linked under a shared experimental-program identifier without merging publication records. Do Carmo et al. [44,45], Laporta et al. (2014) [46], Paradizo et al. (2024) [47], and Vivián et al. (2026) [48] were identified as related outputs of the Bernardo Rosengurtt Angus–Hereford herbage-allowance program in Uruguay. The two Do Carmo publications report complementary outcomes from the 2007–2010 experiment; Laporta et al. (2014) and Paradizo et al. (2024) evaluated physiological and reproductive variables in 32-cow subsets during the earlier program [46,47]; Vivián et al. (2026) analyzed reproductive events from the continuing 2007–2022 program [48]. Precise animal-level overlap cannot be reconstructed from available identifiers, so these records were not treated as independent replications. The Claramunt studies [49,50] arose from a separate primiparous Hereford grazing program and were not assigned to the Bernardo Rosengurtt cohort. Supplementary Table S1 summarizes the available search-term combinations and aggregate database returns. However, complete database-specific executed search histories, including field restrictions, search limits, execution dates, and intermediate modifications, were not preserved. Consequently, Supplementary Table S1 documents the available search strategy but does not constitute a fully reproducible record of every executed query. Aggregate retrieval counts were not interpreted as unique screened records, and retrospective deduplication or exclusion counts were not inferred. These limitations should be considered when evaluating the comprehensiveness and reproducibility of the evidence inventory. The inventory was assembled iteratively from studies identified through database searching, reference checking, and manuscript-directed citation reconciliation. Records S028–S035 were added. S036 (Laporta et al. 2014, [46]) was incorporated during October 2026 reconciliation after the 16 September 2026 search cut-off; its later entry date is not a retrospective extension of the database search. The Level I–IV totals describe the final discussed-publication inventory only.

### 2.4. Classification of Evidence

Because studies differed substantially in design, genotype, nutritional exposure, and reproductive outcome, evidence was classified according to its directness to the central G × N question, rather than treated as equivalent. Classification was as follows:

Level I—Direct reproductive G × N evidence: Studies comparing two or more genotypes under contrasting nutritional, HA, stocking, or grazing environments in a factorial or equivalent analytical framework and directly evaluating G × N for a reproductive, maternal, calf-survival, or cumulative reproductive cow–calf performance endpoint. Factorial genotype × nutritional-environment studies limited to intake, metabolic, physiological, or biological-efficiency endpoints were not classified as Level I, even when informative mechanistically.

Level II—Genotype and crossbreeding evidence: Studies evaluating genotype, crossbreeding, maternal heterosis, reproductive performance, survival, longevity, or cow–calf productivity without a controlled nutritional comparison [51,52,53,54,55,56,57,58,59,60,61,62].

Level III—Nutritional and grazing-management evidence: Studies directly evaluating HA, forage availability, stocking rate, supplementation, body condition, or nutritional status in relation to reproductive performance without direct genotype comparison [49,63].

Level IV—Mechanistic and supportive evidence: Mechanistic, physiological, metabolic, endocrine, genomic, behavioral, modeling, review, broader genotype × environment, or other supportive evidence informing biological interpretation without directly testing G × N for a reproductive, maternal, calf-survival, or cumulative reproductive cow–calf performance endpoint. This level includes factorial genotype × nutritional-environment studies whose evaluated endpoints are limited primarily to intake, physiology, or biological efficiency.

Accordingly, factorial genotype × nutritional-environment studies evaluating mechanistic or physiological endpoints without direct reproductive or cow–calf performance outcomes were classified as Level IV rather than Level I. Similarly, studies demonstrating genotype × broader-environment interactions without an experimentally defined nutritional contrast were considered supportive evidence rather than direct reproductive G × N evidence.

This hierarchy describes directness to the G × N question rather than study quality or certainty. For Level I, the analysis must specify a genotype × nutritional-environment term for an observed reproductive endpoint; statistical significance is not required, and an unreported interaction estimate must not be construed as a positive result. Laporta et al. (2014) [46] modeled genotype × herbage allowance for postpartum commencement of luteal activity but did not separately report that reproductive interaction result; Vivián et al. (2026) [48] reported a stage-specific reproductive interaction. Under the primary analysis, both are Level I, whereas a stricter sensitivity rule requiring a separately reported reproductive interaction estimate or test classifies Laporta et al. (2014) [46] as Level IV (Level I = 1; Level II = 14; Level III = 9; Level IV = 12). Claramunt et al. (2020) [50] is Level IV because it reports endocrine, behavioral, and calf-production endpoints without a genotype comparison or direct reproductive outcome; Claramunt et al. (2024) [49] and Briano et al. (2024) [63] are Level III because they report reproductive outcomes under nutritional treatments without a breed/genotype × nutrition test. Parity × nutrition is not equivalent to breed/genotype × nutrition.

### 2.5. Evidence Synthesis

Evidence was synthesized across three sequential reproductive stages: (I) breeding to pregnancy diagnosis, encompassing postpartum reproductive recovery, conception, and early pregnancy establishment [2,9,10]; (II) pregnancy diagnosis to calving, encompassing pregnancy maintenance and fetal development [5,12]; and (III) calving to weaning, incorporating maternal performance, calf survival, and preweaning performance [20,21]. Calves weaned per cow exposed and, where available, kilograms of calf weaned per cow exposed were considered cumulative endpoints integrating losses across these stages [3,20,21].

Within each stage, evidence was evaluated for genotype, nutritional environment, and G × N effects, with attention to breed composition, parity, body condition, lactational demand, climatic stress, and previous nutritional exposure [24]. Greater interpretive weight was assigned to studies directly testing G × N and to reproductive outcomes most applicable to grazing beef cow–calf systems.

A formal meta-analysis was not undertaken because of substantial heterogeneity in breed composition, nutritional treatments and HA definitions, physiological stage, production environment, management, and reproductive outcome definitions. Findings were instead critically synthesized according to directness, consistency, magnitude, biological plausibility, and applicability to grazing cow–calf systems.

Of 36 publication-level records, two met the direct reproductive G × N analysis criterion (Level I). Only Vivián et al. (2026) [48] reported evidence of a positive stage-specific interaction; Laporta et al. (2014) [46] included the interaction in the stated reproductive model but did not separately report its estimate or *p*-value. Both originated from the Bernardo Rosengurtt experimental program and are not independent replications. Independent genotype and nutritional main effects are better documented than reproducible reproductive interactions.

The 36-record evidence inventory comprised Level I (*n* = 2), Level II (*n* = 14), Level III (*n* = 9), and Level IV (*n* = 11). Counts describe publications, not independent experiments. Database-search aggregate returns and limitations are reported in Supplementary Table S1; Supplementary Figure S1 illustrates evidence assembly without unsupported retrospective PRISMA attrition counts.

## 3. Systems, Nutritional and Genetic Frameworks, and Measurement of Reproductive Performance

### 3.1. Production Systems

Research on reproductive performance in grazing cattle encompasses diverse environmental, biological, and management conditions [38]. Relevant systems include extensive beef production in Uruguay, Brazil, and Argentina; semi-arid grazing systems in Australia; rangeland-based beef production in the United States; and seasonal pasture-based dairy systems in New Zealand [39]. Although these systems differ in production objectives and management intensity, all depend substantially on grazed forage and face seasonal and interannual variation in forage supply.

South American beef production commonly relies on native grasslands with relatively low external inputs and substantial climatic variability [40]. Comparable nutritional challenges occur in semi-arid and subtropical Australian systems, where variable rainfall and drought can constrain forage availability and animal performance [41]. U.S. rangeland beef systems likewise experience seasonal variation in forage quantity and quality, although supplementation is often used to mitigate nutritional deficits [42]. In contrast, New Zealand dairy systems represent more intensively managed pasture-based production, with seasonal calving designed to align lactational demand with pasture growth [64,65].

Despite these differences, variability in climate, pasture growth, and animal responses is common in grazing systems [41,66]. Longitudinal and factorial studies incorporating genetic and nutritional treatments are therefore particularly valuable for distinguishing genotype and environmental main effects from their interactions [67] and for evaluating performance across years and production cycles [68].

### 3.2. Nutritional Management and Herbage Allowance

Nutritional management is a major determinant of reproductive performance in grazing cattle, and herbage allowance (HA) provides a useful framework for relating forage supply to animal demand [69]. HA is generally expressed as forage dry matter available relative to animal live weight over a defined period and integrates pasture biomass, stocking rate, and grazing management [70]. As HA declines, restrictions on forage selection and intake can reduce energy availability; at greater HA, intake and individual-animal performance generally increase until additional forage availability produces diminishing responses [71].

In extensive beef systems, HA is commonly manipulated through stocking rate [44]. Lower HA increases grazing pressure and can restrict intake and dietary selection, contributing to unfavorable energy balance and loss of body reserves. Greater HA generally increases opportunities to select higher-quality forage and can support improved nutritional status and reproductive performance. Importantly, however, simultaneous effects of genotype and HA do not establish a G × N interaction unless genotype responses differ across nutritional environments [44].

The nutritional environment experienced by grazing cattle also depends on spatial and environmental factors. Terrain, distance to water, soil characteristics, and pasture heterogeneity can influence grazing distribution and access to forage [72,73,74,75,76]. Pasture mineral composition can also vary and may affect the adequacy of mineral supply to grazing livestock [77]. Consequently, measured pasture biomass or nominal HA may not fully represent the functional forage availability experienced by individual animals.

Supplementation can mitigate seasonal nutritional deficits in rangeland systems [78], whereas feed allowance and pasture management play analogous roles in seasonal pasture-based dairy production [79]. Across systems, the principal biological pathway is similar: forage availability influences nutrient intake, energy balance, body reserves, and metabolic–endocrine status, with downstream effects on ovarian activity, embryo development, pregnancy maintenance, lactation, and maternal performance [80,81]. Thus, HA provides both a management variable and an environmental framework for evaluating nutritional effects on cumulative reproductive efficiency [45].

### 3.3. Crossbreeding, Heterosis, and Genetic Strategies

Crossbreeding is widely used to exploit heterosis and breed complementarity, particularly for low-heritability fitness and maternal traits. In beef systems, combinations such as Hereford × Angus illustrate how complementary breed characteristics and heterosis can improve maternal and productive performance [18,21,22,51]. Pasture-based dairy crossbreeding, including Holstein–Friesian × Jersey populations, provides comparative evidence for heterosis-related advantages in fertility and survival [23]. However, these outcomes are used here as supportive rather than directly equivalent evidence for beef cow–calf systems.

Evidence from crossbreeding studies indicates that maternal heterosis can improve reproductive performance and calf productivity, although the magnitude and statistical significance of these benefits vary among breeds, traits, and production systems. Neville et al. (1984) [52] reported generally positive but statistically nonsignificant heterosis estimates for reproductive traits in rotationally crossbred beef cattle. In contrast, Kress et al. (1990) [51] documented substantial maternal heterosis for calving proportion and calf weaning weight per cow exposed, particularly among two-year-old dams. Collectively, these findings support the biological importance of heterosis for reproductive and maternal traits while emphasizing that its expression is context-dependent. However, these studies did not directly test genotype × nutritional environment interactions, and their findings should not be interpreted as evidence of such interactions.

Interpretation requires distinction among crossbreeding systems. F1 females generally express high heterozygosity, whereas rotational systems retain varying proportions of heterosis across generations, and terminal systems emphasize performance of market offspring rather than replacement females. Thus, “crossbred” should not be treated as a single genotype; breed composition, generation, retained heterozygosity, direct and maternal heterosis, and breed complementarity may all influence reproductive and cow–calf performance.

Because reproductive traits generally have relatively low heritability, non-additive genetic effects can be biologically important. Nevertheless, superior average performance of a crossbred group does not demonstrate G × N. Such inference requires evidence that the magnitude or direction of genotype-associated performance differs across nutritional environments. These distinctions are examined in detail in Section 4.2 and Section 4.5.

### 3.4. Measurement of Reproductive Performance

Accurately defining reproductive outcomes is essential because commonly reported measures differ in their denominators, observation periods, and biological interpretation. In beef cow–calf systems, reproductive performance can be considered across three sequential stages: (I) breeding to pregnancy diagnosis, incorporating conception and early pregnancy establishment; (II) pregnancy to calving, reflecting pregnancy maintenance and fetal survival; and (III) calving to weaning, incorporating maternal performance and calf survival [82].

Stage-specific outcomes identify where reproductive losses occur, whereas cumulative measures integrate losses across the production cycle. Calves weaned per cow exposed to breeding is particularly informative because it incorporates failure to conceive, pregnancy loss, calving failure, and preweaning calf mortality [83]. Kilograms of calf weaned per cow exposed extends this measure by incorporating calf growth and therefore more directly represents saleable biological output.

Pasture-based dairy systems use related but distinct measures, including submission rate, conception rate, six-week in-calf rate, and calving interval [84,85]. These outcomes provide comparative information on fertility in forage-dependent systems but should not be considered equivalent to cumulative beef cow–calf endpoints.

Accordingly, this review evaluates reproductive performance across the breeding-to-pregnancy, pregnancy-to-calving, and calving-to-weaning continuum, using cumulative outcomes to integrate losses across successive stages. Table 1 defines and interprets the principal outcomes used throughout the evidence synthesis.

Particular caution is required when comparing studies because pregnancy, conception, calving, and weaning rates may use different denominators and should not be interpreted as equivalent measures of reproductive efficiency. For beef cow–calf systems, calves weaned per cow exposed provides an integrated measure of reproductive success, whereas kilograms of calf weaned per cow exposed additionally incorporates preweaning growth and more directly reflects saleable biological output.

### 3.5. Robustness, Resilience, and Adaptability

The terms robustness, resilience, and adaptability describe related but distinct responses to environmental variation. **Robustness** refers to the capacity of an animal or genotype to maintain biological or productive function across a range of environmental conditions, including variation in nutrient availability. **Resilience** refers more specifically to the capacity to withstand a disturbance and/or recover function thereafter and therefore ideally requires longitudinal measurements of both performance decline and recovery. **Adaptability** describes a genotype’s suitability for, or capacity to function effectively within, a particular environmental or production context.

Within the evidence reviewed here, robustness is generally the most appropriate term for comparing genotype responses across contrasting HA. A smaller decline in reproductive or maternal performance as nutritional conditions deteriorate would indicate greater nutritional robustness. In contrast, most available studies do not quantify recovery trajectories following restriction and therefore provide limited direct evidence of resilience. Likewise, superior performance of a genotype within a particular environment may indicate adaptability but does not necessarily demonstrate robustness across environments or resilience following nutritional challenge.

The classified study-level evidence synthesis draws on the 36-record evidence pool documented in Data File S1. The available literature differs substantially in inferential strength. Relatively few records directly test genotype × nutritional environment (G × N) relationships for reproductive or cow–calf outcomes, whereas a broader body of genotype/crossbreeding, nutritional, mechanistic, and contextual evidence informs interpretation. Evidence of genotype and nutritional main effects is not interpreted as evidence of interaction unless genotype responses differ across nutritional environments.

## 4. Synthesis of Evidence

### 4.1. Genotype and Heterosis Effects

Evidence for genotype-associated reproductive and maternal-performance differences is substantially broader than evidence directly demonstrating G × N. Across grazing and pasture-based cattle systems, crossbreeding is generally associated with favorable reproductive, maternal, survival, and lifetime-performance traits, although responses vary with breed composition, production environment, physiological stage, and outcome [21,22,23,27,51,86,87].

The Level II evidence inventory adds several classical crossbreeding records that reinforce the breadth and context-dependence of Level II evidence. Neville et al. evaluated reproductive performance and calf performance in grading and rotational systems [52,53]; Gotti et al. (1985) [54] reported reproductive outcomes in Angus, Santa Gertrudis, and Gelbvieh crosses; and two studies by Williams et al. evaluated reproductive traits and genetic effects across two-, three-, and four-breed rotational systems [55,56]. The Kress series further documented maternal heterosis, reproduction, calf output, and cow productivity across Hereford-, Angus-, and Simmental-derived groups [51,57,58,59,60]. Dearborn et al. (1987) quantified maternal heterosis for preweaning traits [61], Cundiff et al. (1992) demonstrated cumulative lifetime-production effects [30], and Wetlesen et al. (2022) extended breed and heterosis evaluation to commercial suckler-cow productivity and economic traits [62]. Collectively, these records support genotype and maternal-heterosis effects but do not, by themselves, establish nutritional G × N.

Reproductive and fitness traits generally have relatively low heritability and may therefore benefit from non-additive genetic effects [18,27,86]. Crossbreeding exploits individual heterosis and breed complementarity, while cow–calf performance can additionally benefit from maternal heterosis. These effects may be expressed through fertility, calf survival, maternal ability, longevity, and cumulative calf production rather than pregnancy rate alone. Studies involving Hereford, Angus, Simmental, Brangus, Nelore, Caracu, and other breed combinations demonstrate substantial variation in heterosis among traits and genetic backgrounds [20,21,51,86].

Estimates of reproductive and maternal heterosis are commonly favorable, but their magnitude should not be generalized across breeds, ages, traits, or production systems [51,52,53,58,62]. Maternal heterosis may be particularly important for cumulative cow–calf productivity because advantages expressed in fertility, maternal performance, calf survival, longevity, and herd retention can accumulate across production cycles [30,87]. These studies do not establish genotype × nutritional-environment interactions.

The age-specific estimates reported by Kress and colleagues illustrate why reproductive heterosis should be expressed with its original denominator and scale. Among two-year-old cows, Kress et al. (1990) [51] estimated maternal heterosis of 43.1% for the proportion calving and 34.6% for calf weaning weight per cow exposed. In a related analysis of cows aged three to eight years, Kress et al. (1992) [58] estimated maternal heterosis of 11.5% for the proportion calving, 10.4% for the proportion weaning a calf, and 17.9% for calf weaning weight per cow exposed. These values are relative percentage heterosis estimates, not absolute percentage-point increases. The two reports represent related research cohorts with different age structures and endpoints; they should not be interpreted as independent replication or as evidence of a nutritional interaction.

Pasture-based dairy studies provide complementary evidence for heterosis-related fertility and survival advantages [23]. However, dairy endpoints such as submission rate, six-week in-calf rate, and calving interval are not equivalent to calves weaned per cow exposed. Dairy evidence is therefore used here to assess the consistency of broader biological relationships rather than to estimate crossbreeding effects in beef cow–calf systems.

### 4.2. Components of Crossbreeding Effects: Individual Heterosis, Maternal Heterosis, and Breed Complementarity

Interpreting crossbreeding effects requires distinguishing among individual heterosis, maternal heterosis, and breed complementarity. Classifying as simply purebred or crossbred can obscure variation in breed composition, heterozygosity, generation, and mating system.

Individual heterosis reflects performance associated with heterozygosity in the offspring relative to the parental-breed average, whereas maternal heterosis reflects advantages attributable to the crossbred dam. The latter can influence fertility, maternal performance, calf survival, longevity, and ultimately, cumulative cow–calf output. Breed complementarity is distinct from heterosis and reflects the combination of advantageous characteristics from component breeds.

For calves weaned per cow exposed, maternal heterosis is particularly relevant because successful weaning depends on pregnancy establishment and maintenance, calving, lactation, maternal ability, calf survival, and the cow’s retention in the breeding herd. Modest advantages at several stages can therefore accumulate into larger differences in cumulative productivity.

The amount of heterosis retained also depends on mating design. F1 crosses generally maximize heterozygosity between parental breeds, whereas rotational and later-generation systems retain different proportions. Consequently, G × N responses observed in one crossbreeding system should not automatically be generalized to others. Apparent nutritional robustness may reflect heterosis, breed complementarity, mature size, milk potential, or combinations of these factors, reinforcing the importance of reporting breed composition and crossbreeding structure.

### 4.3. Nutritional and Herbage Allowance Effects

Nutritional status is a major determinant of reproductive performance because forage availability and quality influence nutrient intake, energy balance, body condition, metabolic–endocrine function, and progression through successive reproductive stages [88,89,90]. HA therefore provides an important environmental exposure linking pasture conditions with animal nutritional status.

The Level III evidence pool broadens this nutritional foundation across controlled and observational settings. Selk et al. (1988) related prepartum nutrition, weight change, and body condition to reproduction in range cows [91]; Morrison et al. (1999) showed that large prepartum body-condition changes did not impair subsequent reproduction when cows calved in moderate condition [92]; Rae et al. (1993) documented strong associations of parity and BCS with pregnancy in commercial Florida herds [93]; and Mulliniks et al. (2012) [94] found that young cows adapted to native range could maintain high pregnancy rates across a relatively narrow range of calving BCS. Carvalho et al. (2022) associated BCS at calving and post-calving BCS change with FTAI pregnancy in Bos indicus cows [95]. Controlled nutritional studies by Warren et al. (1988) [96] and DeRouen et al. (1994) [97] further demonstrate that reproductive responses depend on initial body condition, timing, and severity of nutritional exposure rather than on a single static BCS threshold.

Importantly, HA represents environmental exposure, whereas BCS and body-reserve change are major animal-level mediators. Greater HA can increase nutrient intake and support energy balance and preservation of body reserves; inadequate HA can produce negative energy balance and mobilize body-reserves. BCS, and particularly, longitudinal BCS change, therefore, provides important phenotypic links between nutritional environment and reproductive performance [88,89].

The principal pathway can be conceptualized as follows:

HA → nutrient intake → energy balance → BCS/body reserves → metabolic–endocrine signaling → reproductive and maternal function.

Adequate nutritional status supports postpartum ovarian activity, conception, pregnancy maintenance, fetal development, lactation, and calf growth [48,49,98]. Conversely, nutritional restriction can delay postpartum cyclicity and adversely affect follicular development, oocyte competence, embryo survival, and subsequent reproductive performance [80,88,89].

Nutritional effects may also persist beyond the restriction period. Experimental grazing evidence indicates that HA during one physiological stage can influence subsequent BCS, metabolic, and reproductive responses [49]. Supplementation may mitigate seasonal deficits, but responses depend on forage quality, severity and duration of restriction, parity, physiological stage, and initial BCS. Nutritional effects should therefore be interpreted within the temporal and physiological context of exposure.

Herbage-allowance studies in the evidence inventory provide complementary physiological and production context. Claramunt et al. (2020) linked contrasting HA with metabolic hormones, grazing behavior, maternal behavior, and productive responses [50], while Briano et al. (2024) reported parity-dependent metabolic, productive, and reproductive responses to late-gestation forage allowance [63]. Together with the direct and carry-over reproductive experiment of Claramunt et al. (2024) [49], these records support HA as a biologically meaningful nutritional exposure while also showing that parity and physiological stage modify the observed response.

### 4.4. Stage-Specific Reproductive Responses

**Figure 1:**
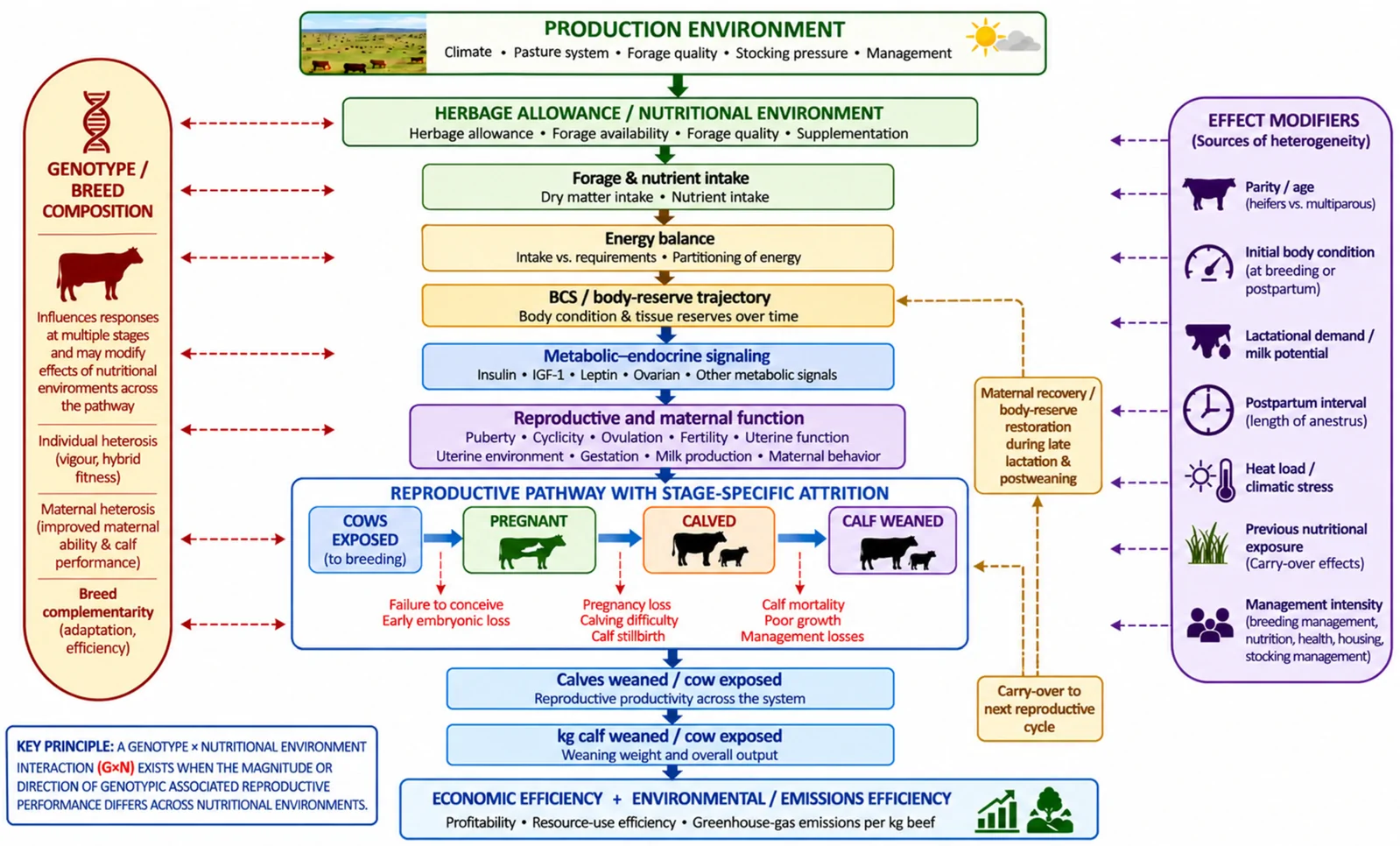
Conceptual framework linking genotype, nutritional environment, stage-specific reproductive attrition, and cumulative cow–calf efficiency in grazing cattle. Herbage allowance, forage quality, stocking pressure, and production-environment features influence forage and nutrient intake, energy balance, and body-condition/body-reserve trajectories, which in turn affect metabolic–endocrine signaling and reproductive and maternal function. Reproductive success progresses through sequential breeding-to-pregnancy, pregnancy-to-calving, and calving-to-weaning stages, with losses at each transition determining cumulative calves weaned per cow exposed and kilograms of calf weaned per cow exposed. Genotype, including breed composition, individual and maternal heterosis, and breed complementarity, may modify responses to nutritional environment across these stages. Parity, initial body condition, postpartum interval, lactational demand, climatic stress, previous nutritional exposure, and management further modify these relationships. The framework distinguishes independent genotype and nutritional effects from a true genotype × nutritional environment interaction, which requires the magnitude or direction of genotype-associated performance to differ across nutritional environments. Breed complementarity is depicted as potential adaptation and efficiency, not demonstrated recovery (resilience) following nutritional restriction. Economic and emissions pathways are conceptual and not direct outcomes quantified by the reproductive G × N studies.

Reproductive losses accumulate across biologically distinct stages between exposure to breeding and weaning. Pregnancy rate alone may therefore underestimate genotype- and nutrition-associated effects on whole-system reproductive efficiency. Here, the reproductive continuum is defined as breeding-to-pregnancy, pregnancy-to-calving, and calving-to-weaning, as summarized conceptually in Figure 1.

The breeding-to-pregnancy stage encompasses postpartum reproductive recovery, ovulation, fertilization, embryo development, and establishment of pregnancy and is particularly sensitive to metabolic and endocrine status [81,88,90]. Nutritional restriction can delay ovarian cyclicity and compromise oocyte, luteal, and early embryonic function [99,100]. Although crossbreeding may improve fertility-related traits, greater average fertility in crossbred females does not establish G × N unless the genotype difference changes with the nutritional environment.

The pregnancy-to-calving stage reflects maintenance of established pregnancy. Nutritional restriction, placental dysfunction, heat stress, disease, and other challenges may compromise gestation, although direct evidence for genotype-dependent responses during this stage remains limited. Proposed mechanisms such as differences in placental or physiological function should therefore be interpreted cautiously [101].

The calving-to-weaning stage integrates maternal performance, lactation, calf vigor and survival, and preweaning growth. Maternal heterosis may be particularly important during this period [20,21,51], and under nutritional restriction, differences in body-reserve management and maternal performance may become increasingly consequential. Grazing studies provide evidence that genotype and HA influence components of cow–calf efficiency and that maternal heterosis can contribute substantially to preweaning output [44,45,98].

Thus, genotype and nutrition may affect different components of reproductive success at different stages, supporting evaluation of the entire breeding-to-weaning continuum rather than pregnancy rate alone.

### 4.5. Evidence for Genotype × Nutritional Environment Interaction

The central question is whether the magnitude or direction of genotype-associated reproductive performance changes across nutritional environments. Genotype and nutrition can each have substantial main effects without a statistically or biologically meaningful interaction.

**Figure 2:**
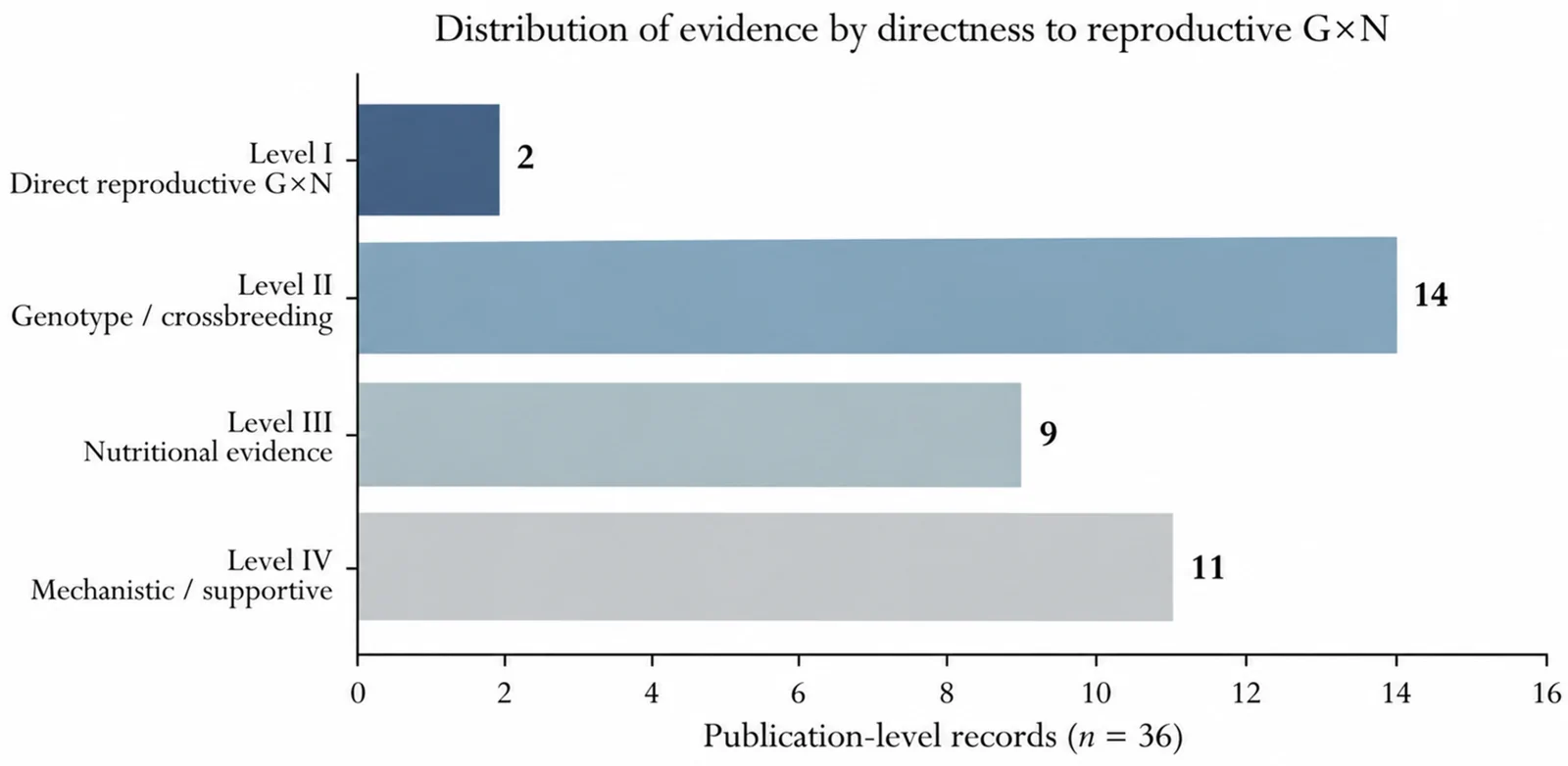
Distribution of 36 publication-level records in Data File S1: Level I, direct reproductive G × N analysis (*n* = 2); Level II, genotype/crossbreeding evidence (*n* = 14); Level III, nutritional evidence (*n* = 9); and Level IV, mechanistic/supportive evidence (*n* = 11). Level I includes one publication with a reported stage-specific interaction (Vivián et al. 2026) and one that specified the interaction model for postpartum luteal activity without separately reporting the interaction result (Laporta et al. 2014) [46,48]. Counts do not represent independent experimental populations.

Among 36 publication-level records, 2 met the prespecified Level I model criterion (Figure 2). Vivián et al. (2026) [48] analyzed 1069 reproductive events (650 purebred and 419 crossbred event records) from Hereford, Angus, and reciprocal F1 cows at the Bernardo Rosengurtt station during 2007–2022. A randomized-block grazing experiment used two blocks and a 2 × 2 genotype × herbage-allowance arrangement; annual target HA was 4.0 versus 2.5 kg forage DM/kg BW for the earlier period, with reported averages of 6.0 versus 3.0 kg DM/kg BW for 2015–2022. Allowance was managed by pasture stocking adjustments. Bayesian Bernoulli–logit mixed models included genotype, HA and their interaction, with cow identity and year random effects. Event totals are not independent animal sample sizes; unique cow counts and genotype-by-HA cell counts were not explicitly reported in the provided paper. Laporta et al. (2014) [46] studied 32 multiparous Angus, Hereford, and reciprocal F1 cows in May 2009–March 2010, with 4.0 versus 2.5 kg DM/kg BW annual target HA. Their randomized-block factorial experiment had two blocks and four grazing plots per block, with cows maintained within plots and longitudinal metabolic, endocrine, and reproductive observations. Their article includes genotype × HA in its model for postpartum commencement of luteal activity but does not provide a separately identifiable interaction estimate for that endpoint. Neither study supplies an independent geographic replication of reproductive G × N.

Evidence from Levels II–IV should not be interpreted as direct reproductive G × N replication. Level I denotes an explicit reproductive interaction analysis, not necessarily a significant or independently replicated interaction.

Vivián et al. (2026) [48], reports calving-to-weaning posterior probabilities for purebred versus crossbred cows of 0.67 versus 0.90 under low HA and 0.79 versus 0.88 under high HA, with 95% HPDI intervals shown in the source. Further. it reports within-HA genotype contrasts for Period III of 0.22450 (95% HPDI 0.06352–0.39997; posterior P[Δ > 0] = 1.00000) under low HA and 0.09506 (95% HPDI 0.00335–0.20440; posterior P[Δ > 0] = 0.99050) under high HA. These are within-environment genotype contrasts, not a separately tabulated posterior difference-in-differences interaction interval. The reported pattern is specific to the Bernardo Rosengurtt program.

For interpretation of the Bayesian results, the genotype contrast within a given herbage-allowance treatment should be distinguished from the interaction contrast between treatments. A larger crossbred–purebred difference under low than under high herbage allowance is descriptively compatible with nutritional robustness, but the numerical difference between two rounded contrasts is not itself a separately estimated posterior genotype × herbage-allowance effect. The strongest direct inference remains the stage-specific reproductive response reported by Vivián et al. (2026) [48], interpreted within the limitations of a single long-term experimental program. Laporta et al. (2014) [46] also meets the prespecified Level I model-based classification, but its unreported reproductive interaction estimate cannot be counted as a positive finding.

The remaining records in Data File S1 should therefore be interpreted as triangulating evidence rather than additional direct replication. Classical crossbreeding cohorts provide Level II evidence of genotype and maternal-heterosis effects without controlled nutritional comparisons [30,51,52,53,58,61,62]. Nutritional reproductive studies, including Claramunt et al. (2024) [49], provide Level III evidence, whereas intake, physiological, and other mechanistic studies, including Do Carmo et al. [44,45], Claramunt et al. (2020) [50], and Paradizo et al. (2024) [47], provide Level IV support. These examples do not constitute an exhaustive citation-by-level inventory; the publication-level assignments are documented in Data File S1. Franke’s synthesis of American Zebu breed and heterosis effects provides historical context [102], and the meta-analysis by Gonçalves et al. (2025) provides contextual evidence on supplementation and grazing cow–calf performance [103]. Neither synthesis constitutes independent direct reproductive G × N replication. Kress et al. (1990) [51] reports 264 two-year-old dams and maternal heterosis of 43.1% for proportion calving and 34.6% for calf weaning weight per cow exposed (abstract, p. 54); Kress et al. (1992) [58] reports 766 breeding exposures, 581 calves, and 11.5%, 10.4% and 17.9% maternal heterosis for proportion calving, proportion weaning and calf weaning weight per cow exposed, respectively (p. 2682). Ribeiro et al. (2022) [104] addresses body condition and mature weight, not direct reproductive G × N. Bunning et al. (2019) [105] conducted a meta-analysis of 42 studies comprising 518 heterosis estimates in tropical cattle. Fertility heterosis was estimated at 12.02 ± 4.10% (least-squares mean ± standard error; 40 fertility estimates; p. 31). Although trait category and breed combination significantly influenced heterosis, the effect of climate was not statistically significant. This synthesis provides contextual evidence for crossbreeding benefits but does not directly test genotype × nutritional-allowance interactions.

Do Carmo et al. (2018) [45] examined the 2007–2010 Campos grazing program, including cow and calf production outcomes. The related Do Carmo et al. (2021) [44] analyzed herbage intake and biological efficiency in the same program, not an independent reproductive cohort. The 2021 randomized complete-block design comprised two blocks and eight grazing plots in a 2 × 2 herbage-allowance (annual mean high approximately 5 vs. low 3 kg DM/kg BW) × genotype (straightbred Hereford/Angus vs. reciprocal F1 crossbred) factorial; 24–36 cows were sampled across six intake-measurement periods. The crossbred versus straightbred mid-late gestational DMI was 81 versus 94 g/kg metabolic BW/day (genotype *p* = 0.05), and high- versus low-allowance lactational DMI was 115.6 versus 94.1 (allowance *p* < 0.05). No significant HA × genotype interaction for DMI was detected (Methods and Results, pp. 5–6). Further, it reports biological efficiency of 40.0 versus 26.2 g calf/kg DMI for crossbred versus straightbred cows (*p* = 0.05) and 36.0 versus 29.7 for high versus low allowance (*p* < 0.05). These are marginal treatment comparisons and should not be interpreted as evidence of a significant genotype × allowance interaction for reproductive success. The biological-efficiency numerator used a 200-day simulated calf weaning weight multiplied by treatment-specific weaning rates from an earlier study, rather than independently observed reproductive outcomes in this intake sample (Methods, p. 4).

Broader genotype × environment evidence is heterogeneous across cattle populations. Morris et al. (1993) [106] analyzed 7575 mating records from 2109 cows across contrasting New Zealand locations and reported genotype × environment interactions for reproductive and maternal components, particularly calf weight weaned per cow joined and cow efficiency. In contrast, Jeyaruban et al. (2009) [43] estimated between-state genetic correlations of 0.97–1.00 for reproductive traits in Australian Angus cattle from Victoria and Queensland, indicating little evidence of G × E for those traits. The studies differ in breed composition, environmental contrast, and analytical approach. Neither experimentally isolated nutritional exposure; therefore, both provide broader environmental context rather than direct reproductive G × N evidence.

Studies addressing individual components of the proposed interaction provide supportive evidence. Winder et al. (1992) [98] reported substantial maternal heterosis in cows maintained under semidesert conditions, but nutrition was not experimentally manipulated. Andresen et al. (2020) [107] found that crossbred cows maintained greater BCS and consumed less low-quality forage than Angus cows; however, maintenance requirements and preweaning calf-growth efficiency did not differ between breed types. Claramunt et al. (2024) [49] demonstrated direct and carry-over effects of HA on metabolic and reproductive responses but did not compare genotypes. Additional mechanistic evidence from the same broader research program showed that HA and cow genotype influenced follicular, oocyte, and uterine characteristics in purebred and crossbred beef cows [47]. Because reproductive success itself was not the primary endpoint, this study provides mechanistic support for biologically plausible G × N rather than independent confirmation of reproductive-performance G × N. Collectively, these studies support the biological plausibility of genotype-associated nutritional robustness but cannot independently establish reproductive G × N.

Modeling evidence also illustrates current limitations. Elkins et al. (2026) [108] found that the Beef Cattle Systems Model did not reproduce empirically observed genotype-dependent nutritional responses; simulated genotypes responded similarly across DMI levels. This result does not argue against biological G × N but indicates that average-cow nutritional equations may inadequately represent genotype-specific responses.

The only reported positive reproductive G × N finding in this inventory comes from the Vivián et al. (2026) [48] study of the long-term Bernardo Rosengurtt program. The evidence does not establish a universal interaction across breeds, reproductive stages, or grazing environments. A smaller decline in reproductive output under nutritional restriction is consistent with nutritional robustness, not necessarily physiological resilience, because recovery following restriction was not measured.

For cow–calf systems, G × N may ultimately be expressed most strongly through cumulative maternal performance. Relatively small genotype-associated differences in body-condition maintenance, lactation, maternal ability, calf survival, and reproductive persistence may accumulate across the cycle and become more evident in calves weaned per cow exposed.

**Figure 3:**
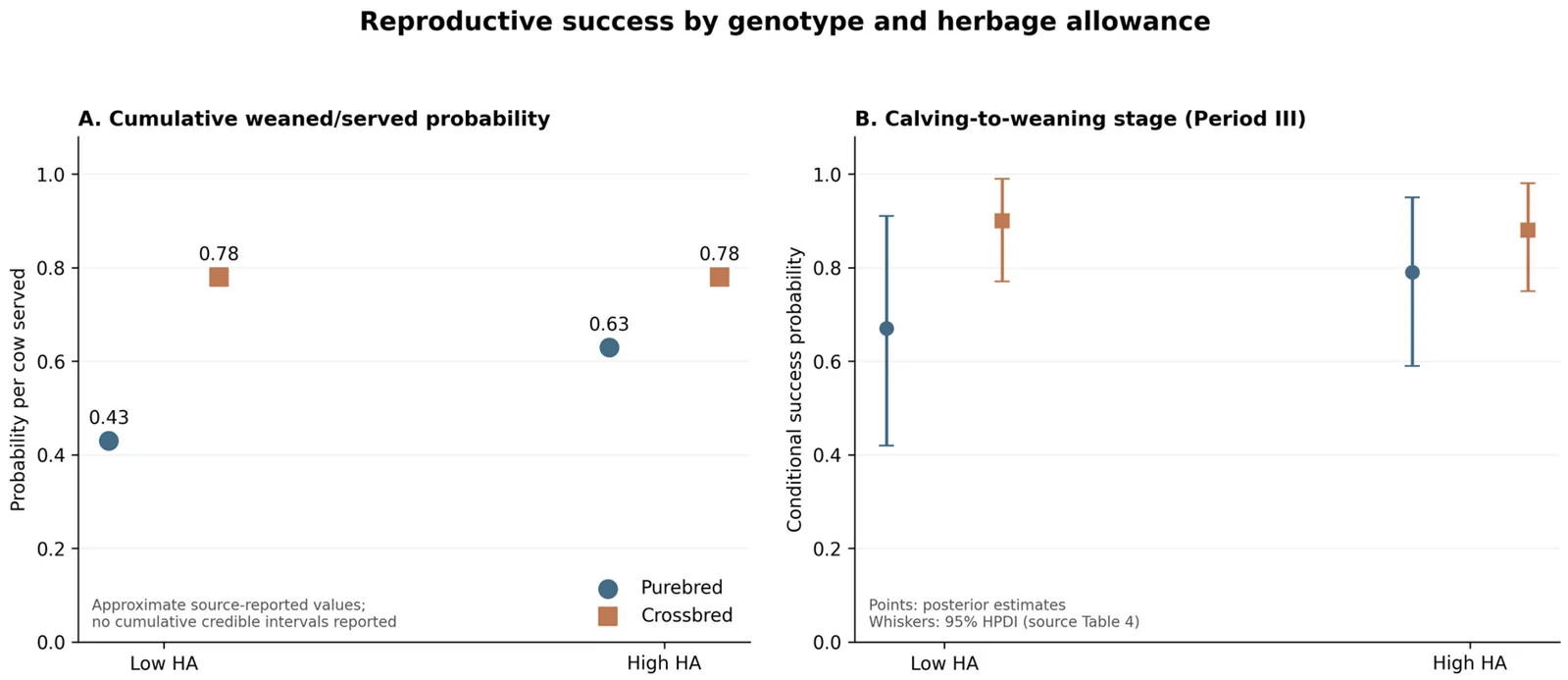
Genotype and herbage-allowance contrasts reported by Vivián et al. (2026) [48]. (**A**) Approximate cumulative model-derived probability of weaning a calf per cow served (weaned/served), as described in the original publication. These values are rounded narrative estimates, and cumulative credible intervals were not reported. (**B**) Posterior probabilities of calving-to-weaning success (Period III), with 95% highest posterior density intervals, as reported in the original publication. The stage-specific estimates in panel B are conditional probabilities and must not be interpreted as cumulative weaned/served outcomes. Points represent discrete low- and high-herbage-allowance treatments rather than a continuous dose-response. The original publication also reports within-allowance genotype contrasts for Period III; however, the plotted intervals represent group-specific probabilities rather than interaction contrasts.

Collectively, these findings suggest that genotype-associated differences in reproductive and cow–calf performance may vary across nutritional environments, although the magnitude and direction of these responses are context- and stage-dependent. Figure 3 depicts the two model-derived herbage-allowance conditions reported by Vivián et al. (2026) [48]. The figure illustrates a smaller decline in estimated cumulative reproductive success among crossbred cows in that experimental population; it does not establish a continuous response curve or generalize this pattern to other breeds.

The heterogeneity of breeds, crossbreeding systems, HA definitions, production environments, climatic exposures, and reproductive endpoints precludes treating these supportive studies as a quantitatively comparable evidence set.

A further limitation is the lack of independent geographic and research-group replication of direct reproductive G × N evidence. The Level I publications arise from a shared Uruguayan grazing program, whereas the Level III nutritional evidence spans several regions and programs and must not be characterized as one Campos cohort. Independent factorial replication across regions and breeds remains necessary.

The distinction between direct reproductive G × N analyses and supporting mechanistic evidence is essential when interpreting factorial grazing studies. Among the 36 publication-level records, two met the predefined Level I eligibility criteria. Vivián et al. (2026) [48] directly evaluated genotype × herbage allowance effects on stage-specific and cumulative reproductive success and reported a positive stage-specific interaction. Laporta et al. (2014) [46] specified a genotype × herbage allowance model for postpartum commencement of luteal activity but did not separately report the interaction estimate or its statistical significance. Thus, although both publications qualify for Level I classification, only Vivián et al. (2026) provided a reported positive reproductive G × N finding. In contrast, two studies by Do Carmo et al. [44,45] and a study by Paradizo et al. (2024) [47] investigated intake, biological efficiency, or reproductive physiology without directly reporting reproductive-success G × N effects. These studies provide complementary mechanistic evidence but should not be interpreted as independent confirmation of reproductive G × N.

#### Independence of Experimental Evidence

An important consideration in interpreting the available G × N evidence is the nonindependence of several publications arising from the same experimental program. The studies reported by Do Carmo et al. [44,45], Paradizo et al. (2024) [47], and Vivián et al. (2026) [48] originated from the long-term Angus–Hereford crossbreeding and herbage-allowance research program at the Bernardo Rosengurtt Experimental Station in Uruguay. The two Do Carmo publications [44,45] reported complementary outcomes from the same 2007–2010 grazing experiment, whereas Paradizo et al. (2024) [47] evaluated follicular and uterine characteristics in 32 cows selected from the earlier experimental population. Vivián et al. (2026) [48] subsequently analyzed 1069 reproductive events collected during 2007–2022 from the continuing experimental program. Although these publications provide complementary physiological, productive, and reproductive evidence, they do not constitute independent experimental replications across distinct populations. Furthermore, the Do Carmo studies [44,45] and Paradizo et al. (2024) study [47] primarily evaluated mechanistic or biological-efficiency outcomes rather than direct reproductive G × N effects. Accordingly, Laporta et al. (2014) [46] and Vivián et al. (2026) [48] constitute the two Level I publications in the present evidence inventory, although only Vivián et al. (2026) [48] reported a positive stage-specific reproductive G × N interaction. Both originated from the same long-term experimental program and therefore do not represent independent geographic or research-group replication. The exact extent of animal-level overlap among publications could not be established from the available reports. Consequently, the reported reproductive G × N response should be interpreted as evidence from one experimental setting rather than as an independently replicated finding. The 2021 Do Carmo et al. [45] intake analysis explicitly referenced the earlier Do Carmo et al. 2018 [45] program and used the same 2007–2010 experimental site; their study populations must not be treated as independent replications.

### 4.6. Modifiers of the Genotype × Nutritional Environment Response

G × N responses are unlikely to be uniform among cows exposed to similar HA. Important effect modifiers include parity and age, initial BCS and body-reserve trajectory, postpartum interval, lactational demand, breed composition, climatic stress, and previous nutritional history.

#### 4.6.1. Parity and Age

Primiparous cows are particularly susceptible to nutritional restriction because nutrients must support maintenance, continued growth, lactation, body-reserve restoration, and reproduction simultaneously. Nutritional exposure in primiparous grazing cows can alter metabolic and reproductive responses, including effects persisting beyond the treatment period [49]. Results obtained in mature cows should therefore not automatically be extrapolated to younger females.

#### 4.6.2. Body Condition and Body-Reserve Dynamics

Initial BCS influences the capacity to buffer nutritional restriction [86,88,89,90,109], but BCS trajectory may be as important as a single measurement. BCS can act as both a mediator of previous nutritional exposure and a modifier of response to subsequent restriction. Studies should therefore distinguish baseline BCS from BCS changes induced by nutritional exposure.

#### 4.6.3. Postpartum Interval and Timing of Calving

Early postpartum cows experience concurrent demands of lactation, uterine recovery, and resumption of ovarian cyclicity [88,89,90]. Later-calving cows also have less time to resume cyclicity before a subsequent seasonal breeding period. Nutritional restriction may amplify these constraints, making postpartum interval an important potential modifier of genotype-associated responses.

#### 4.6.4. Lactation and Milk Potential

Lactation substantially increases nutrient demand. Greater milk potential can improve calf growth but may increase maternal body-reserve mobilization when forage supply is inadequate. Consequently, greater milk production should not automatically be equated with greater biological efficiency. The relevant grazing phenotype may instead be the capacity to support calf growth while maintaining maternal BCS and subsequent reproductive function.

#### 4.6.5. Breed Type and Genetic Background

Breed composition can modify nutritional responses through differences in mature size, milk potential, climatic adaptation, and other functional characteristics. Bos taurus and Bos indicus-influenced crosses should therefore not be considered biologically equivalent. Likewise, nutritional robustness observed for one crossbreeding system should not automatically be generalized to another.

#### 4.6.6. Climatic Stress and Previous Nutritional History

Heat load can reduce grazing and intake and alter metabolic and reproductive function, potentially intensifying nutritional restriction. Consequently, apparent G × N effects under hot or semiarid conditions may form part of a broader genotype × environment response unless nutritional and climatic exposures are measured separately.

Previous nutritional exposure may also influence responses to current HA. Carry-over effects indicate that cows exposed to the same current nutritional environment may differ in BCS, metabolic status, and reproductive potential because of earlier nutritional history [49]. Longitudinal exposure therefore needs to be considered when interpreting G × N.

Overall, genotype and nutritional environment operate within a dynamic physiological context; these modifiers help explain heterogeneity among studies and should be incorporated into future experimental designs.

### 4.7. Inconsistent, Null, and Context-Dependent Evidence

Genotype-associated reproductive advantages and G × N are not uniformly expressed across studies or outcomes. Crossbreeding should not be expected to improve every reproductive trait, in every breed combination, or under every nutritional environment. Likewise, the presence of genotype and nutritional main effects does not imply interaction.

Do Carmo et al. studies [44,45] investigated genotype and herbage-allowance effects within the same Campos grazing program. The 2021 intake study [44] reported lower gestational intake in crossbred cows and higher lactational intake under high herbage allowance, with no significant genotype × allowance interaction for intake. It also reported improved biological efficiency as separate genotype and allowance comparisons; these findings support mechanistic plausibility but do not constitute an independently demonstrated interaction for breeding-to-weaning reproductive success.

Similarly, Vivián et al. (2026) [48] demonstrated stage dependence: genotype differences were more apparent under low HA and particularly during calving-to-weaning, whereas evidence for differential responses was weaker earlier in the reproductive continuum. Thus, positive genotype effects, nutritional effects, and G × N effects can coexist with null responses for other traits or stages.

Crossbreeding responses also depend on breed composition, mating system, and production environment. Genotype × environment evidence from geographically distinct beef production systems [106] further demonstrates that genotype rankings or relative reproductive and maternal performance may vary among environments, although such G × E effects should not automatically be attributed specifically to nutrition. Greater mature size or milk potential may increase nutritional requirements and, under restricted forage conditions, offset part of an expected reproductive advantage. Furthermore, heterosis varies with heterozygosity, generation, and mating system. Results from F1 females therefore should not automatically be extrapolated to later-generation or rotational populations.

These inconsistent and null findings are biologically informative. They support interpretation of G × N as an outcome-specific and context-dependent response, rather than evidence for a universal crossbred advantage.

Laporta et al. (2014) [46] evaluated postpartum commencement of luteal activity under factorial genotype and herbage-allowance treatments. Although the analysis specified a genotype × herbage-allowance term, its reproductive interaction estimate and *p*-value were not separately reported; earlier luteal activity in high-allowance and crossbred cows therefore represents reported main effects rather than demonstrated G × N effects. Paradizo et al. (2024) [47] evaluated follicular and uterine characteristics in 32 multiparous cows from the related Bernardo Rosengurtt program. Genotype and herbage allowance showed physiological main effects, but genotype × herbage-allowance interactions for the examined follicular-fluid variables were not significant (*p* > 0.20). This does not demonstrate a direct reproductive G × N effect and supports Level IV classification. Do Carmo et al. studies [44,45] likewise reported complementary intake and productive outcomes from one 2007–2010 experiment, with no significant genotype × herbage-allowance interaction for principal dry-matter-intake outcomes. In contrast, Vivián et al. (2026) [48] reported a stage-specific reproductive interaction, most evident from calving to weaning. The shared experimental program and unresolved animal-level overlap preclude treating these publications as independent replication. Separately, Claramunt et al. (2020) [50] examined nutritional effects on endocrine, behavioral, and calf-growth endpoints; Claramunt et al. (2024) [49] examined ovulation and pregnancy under contrasting herbage allowances in Hereford cows; and Briano et al. (2024) [63] compared forage allowance and parity rather than breed/genotype × forage allowance (pregnancy interaction *p* = 0.25).

### 4.8. Carry-Over Effects Across Reproductive Stages and Cycles

Nutritional effects may persist beyond the physiological stage during which exposure occurs. In grazing cattle, nutritional conditions during one period can alter body reserves, metabolic status, reproductive timing, maternal performance, and offspring outcomes during subsequent stages [49,88,89,109].

One important pathway is described as follows:

Precalving nutritional environment → BCS/body reserves at calving → postpartum energy balance and BCS change → metabolic/endocrine status → ovarian recovery → subsequent conception.

A second pathway links gestational nutrition with calf outcomes:

Gestational nutritional environment → maternal metabolic and placental/fetal development → calf viability → preweaning performance → successful weaning.

The strength of evidence differs among components of these pathways, and later calf outcomes should not be attributed directly to HA without appropriate longitudinal measurements.

Reproductive events themselves may also create carry-over effects in seasonal systems, as follows:

Delayed conception → later calving → shorter postpartum interval → altered overlap between lactational demand and forage availability → subsequent conception probability.

Such longitudinal feedback may contribute to cumulative reproductive differences among genotypes. If genotypes differ in body-reserve maintenance during restriction, consequences may persist after HA improves. Demonstrating resilience, however, would require measurement of recovery in BCS, metabolic status, ovarian activity, or reproductive function after the nutritional challenge is removed.

These relationships support longitudinal G × N studies spanning multiple reproductive stages and, where possible, successive production cycles.

### 4.9. Production Environment as Context for G × N Responses

Geographic location alone is not a biologically meaningful explanation for differences among studies. More relevant characteristics include forage type and seasonality, rainfall variability, stocking pressure, supplementation, breed composition, heat load, reproductive seasonality, and management intensity.

South American native-grassland studies provide particularly informative evidence because seasonal HA and stocking pressure can be related directly to reproductive and maternal responses [44,45,48,49]. Semi-arid U.S. rangelands provide a different challenge, in which variable precipitation and extensive management may increase the importance of body-reserve maintenance and maternal performance [98]. However, maternal heterosis observed in such environments should be interpreted as heterosis expressed under environmental challenge unless nutrition was explicitly manipulated.

Subtropical systems introduce additional complexity because nutritional restriction may coincide with heat load, forage-quality changes, parasites, and breed-specific climatic adaptation. Responses of Bos taurus crosses in temperate systems should therefore not automatically be extrapolated to Bos indicus-influenced cattle under subtropical conditions.

Seasonal pasture-based dairy systems provide useful comparative evidence [23]. They share dependence on pasture intake, BCS, energy balance, and timely reproductive recovery but differ substantially from beef systems in milk-production potential, genetic selection, management, and reproductive endpoints. Dairy evidence should therefore remain supportive rather than directly quantitative for beef cow–calf efficiency.

Management can also buffer environmental nutritional variation through supplementation, conserved forage, flexible stocking, pasture allocation, or early weaning. Consequently, nominally similar HA may not impose equivalent physiological challenges among systems. The timing of restriction is equally important; deficits during late gestation, early lactation, or breeding may produce different reproductive consequences.

Thus, apparent consistency across geographic regions should not be interpreted as an invariant response. G × N is more appropriately understood within the nutritional, climatic, genetic, reproductive, and management context in which it occurs. Nutritional and climatic exposures should ideally be quantified separately because low HA, drought, and heat load frequently covary.

### 4.10. Cumulative Reproductive Efficiency: Calves Weaned per Cow Exposed

The ultimate reproductive objective of a beef cow–calf system is the successful production of a calf through weaning. Calves weaned per cow exposed integrates reproductive losses during pregnancy establishment, gestation, calving, neonatal survival, lactation, and the preweaning period [101], thereby providing a more comprehensive measure of reproductive efficiency than pregnancy rate alone. Accordingly, calves weaned per cow exposed is the preferred production-level endpoint in this review. However, study-specific outcomes are reported using their original denominators to ensure accurate interpretation and comparability. For example, Vivián et al. (2026) [48] reported model-derived cumulative probabilities of calves weaned per cow served, which should not be considered directly equivalent to calves weaned per cow exposed.

Because reproductive losses accumulate across successive stages, genotype-associated differences can produce substantial differences in cumulative calf output. Vivián et al. (2026) [48] reported model-derived cumulative probabilities of successfully weaning a calf per cow served of approximately 0.43 and 0.63 for purebred cows under low and high herbage allowance, respectively, compared with approximately 0.78 for crossbred cows under both nutritional conditions. The arithmetic differences between these rounded model-derived probabilities correspond to approximately 0.35 under low herbage allowance and 0.15 under high herbage allowance, or 35 and 15 per 100 cows served on the probability scale; these are not observed counts. The resulting approximate 0.20 difference between genotype contrasts is descriptive, not a separately estimated posterior interaction with its own uncertainty interval. The source denominator was cows served, not necessarily cows exposed as defined elsewhere in this review. These findings are consistent with nutritional robustness in this experimental population but do not establish a universally applicable G × N response.

The approximate cumulative values and their denominator are described in the preceding paragraph and in Figure 3A; stage-specific posterior intervals from Vivián et al. (2026) [48] apply only to conditional Period III outcomes shown in Figure 3B, not to the cumulative products.

Cumulative differences may arise from fertility, pregnancy maintenance, maternal heterosis, calf survival, longevity, or resource-use efficiency. Thus, calves weaned per cow exposed should be interpreted as an integrated biological endpoint rather than a direct measure of conception.

Greater cumulative reproductive efficiency may also improve resource-use efficiency because maintenance requirements of breeding females are distributed across greater saleable calf output. Potential reductions in feed, land, or GHG emissions intensity per unit of calf produced, however, depend on stocking rate, herd size, replacement strategy, and whole-system management.

Overall, the evidence supports evaluating genetic and nutritional management across the complete breeding-to-weaning continuum, rather than using pregnancy rate alone to characterize reproductive efficiency.

### 4.11. Lifetime Reproductive Efficiency and Cow Longevity

Annual calves weaned per cow exposed captures reproductive attrition within a production cycle but does not fully represent a breeding female’s biological contribution over her productive life. Small annual differences in fertility, calf survival, maternal performance, and herd retention can accumulate across successive breeding seasons.

Useful lifetime endpoints therefore include lifetime calves weaned per female entering the breeding herd and cumulative kilograms of calf weaned per female. These measures integrate age at first breeding, annual reproductive success, pregnancy and calf losses, calf growth, reproductive failure, culling, and productive lifespan.

Maternal heterosis may be particularly important at this scale. Its biological and economic contribution may result less from a large increase in annual pregnancy rate than from accumulated improvements in fertility, maternal performance, survival, and longevity. Consequently, annual fertility alone may underestimate maternal heterosis’s contribution to cow–calf efficiency.

Few studies simultaneously evaluate genotype, nutritional environment, reproductive attrition, longevity, and lifetime calf output. Long-term cohorts incorporating annual reproductive outcomes, cow survival, replacement, and cumulative kilograms of calf weaned are therefore needed to determine whether G × N expressed within individual reproductive cycles translates into meaningful lifetime advantages.

## 5. From Nutritional Environment to Reproductive Efficiency: Biological and Production Implications

### 5.1. Reproductive Efficiency, Nutrition, and Crossbreeding

Reproductive efficiency is a major determinant of productivity in beef cow–calf systems because failure at any stage from breeding through weaning reduces saleable calf output. At the same time, resources continue to be allocated to maintenance of the breeding female [104]. Because reproductive losses are cumulative, relatively small improvements at individual stages can translate into meaningful gains in calves weaned per cow exposed.

Nutrition is central to this process. In grazing systems, HA and forage quality influence nutrient intake, energy balance, body condition, and reproductive function [49,80,81,88,89]. Negative energy balance, particularly during the postpartum and lactational periods, can delay ovarian activity, alter endocrine and follicular function, and reduce the probability of conception and pregnancy maintenance [9,10,11,49,80,88,89,99,100]. These responses depend on physiological stage, initial body reserves, lactational demand, and the severity and duration of nutritional restriction [13,23,81].

Crossbreeding represents a potential genetic management strategy through heterosis and breed complementarity. Several studies have reported favorable associations between crossbreeding and reproductive, maternal, survival, and longevity traits in beef and pasture-based dairy cattle [18,21,22,23,51]. However, the magnitude and consistency of these associations vary with breed composition, crossbreeding system, reproductive endpoint, and production environment. Evidence suggesting that genotype-associated reproductive responses may differ across nutritional environments is considerably more limited. In particular, findings from the related Angus–Hereford herbage-allowance studies conducted within the Bernardo Rosengurtt grazing research program in Uruguay suggest that genotype-associated differences in reproductive performance may depend on nutritional conditions and reproductive stage, with reported interaction evidence particularly during the calving-to-weaning period [46,48]. These findings are consistent with the possibility of genotype-dependent nutritional robustness within the conditions evaluated in that program. However, because the relevant publications originate from a shared research program, they do not provide independent experimental replication or establish that crossbred females generally maintain superior reproductive performance under nutritional restriction. Whether comparable responses occur in other breed combinations, grazing environments, or management systems remains uncertain.

Production context is therefore important. Grazing systems differ in forage seasonality, rainfall, stocking pressure, supplementation, breed composition, climatic stress, reproductive seasonality, and management intensity. The principal contrasts relevant to interpretation of G × N are summarized in Table 2.

### 5.2. Biological Mechanisms Linking Nutritional Environment, Genotype, and Reproductive Performance

The biological pathway linking nutritional status with reproductive function is well established, whereas mechanisms explaining differential responses among genotypes remain less certain. This distinction is important when interpreting G × N.

#### 5.2.1. From Herbage Allowance to Reproductive Function

HA and forage quality influence reproductive performance principally through their effects on nutrient intake, energy balance, and body reserves [88,89,90]. BCS therefore represents an important intermediary between the grazing environment and reproductive function rather than an independent causal exposure. Ribeiro et al. (2022) [104] evaluated genetic parameters and heterosis for body condition score (BCS) and mature cow weight in a multibreed beef cattle population. Reported direct heterosis estimates were positive for BCS (0.095 ± 0.034 units) and mature cow weight (21.56 ± 3.53 kg). These findings indicate that crossbreeding may influence maternal body condition and mature size, traits relevant to nutritional requirements and reproductive performance. However, that study did not experimentally compare nutritional environments or estimate reproductive G × N interactions; therefore, it provides genetic and physiological context rather than direct evidence of genotype-specific nutritional robustness.

Changes in energy balance and BCS are accompanied by changes in metabolic signals, including insulin, IGF-1, and leptin, which interact with hypothalamic, pituitary, and ovarian function [88,89,90,100]. Nutritional restriction can alter gonadotropin secretion, follicular development, steroidogenesis, ovulation, and postpartum resumption of ovarian cyclicity. Nutritional status during follicular development may also affect oocyte competence and subsequent embryo development, while inadequate nutrition during early gestation may compromise pregnancy establishment and maintenance [88,89,90].

The principal pathway can therefore be summarized as follows:

HA and forage quality → nutrient intake → energy balance and BCS → metabolic/endocrine signaling → hypothalamic–pituitary–ovarian function → follicular development and ovulation → oocyte competence → conceptus development → pregnancy establishment and maintenance.

The magnitude of these effects depends on parity, initial BCS, lactational demand, physiological stage, duration and severity of restriction, and subsequent nutritional recovery.

#### 5.2.2. Potential Modification by Genotype and Heterosis

Mechanisms through which heterosis modifies this nutritional–reproductive pathway are less firmly established. Non-additive genetic effects and breed complementarity may influence several components of maternal and reproductive performance simultaneously [20,21,51,86,87].

Differences in forage utilization and body-reserve dynamics are plausible contributors. Crossbred cows have differed from purebreds in intake, BCS retention, and biological efficiency in some studies [44,45,107]. However, crossbred cows should not be assumed to have universally lower maintenance requirements. Andresen et al. (2020) [107], for example, reported similar maintenance energy requirements in Angus and Hereford × Angus cows despite greater BCS retention and lower voluntary intake of low-quality forage in crossbred cows.

More favorable nutrient partitioning among maintenance, lactation, body reserves, and reproduction is also plausible but remains insufficiently demonstrated. Longitudinal measurements of intake, body composition, BCS, metabolic hormones, milk production, and reproductive function are needed before differential nutrient partitioning can be considered an established mechanism of heterosis.

#### 5.2.3. Maternal Heterosis and Preweaning Performance

Maternal heterosis provides an additional pathway through which crossbreeding may influence cumulative reproductive efficiency [20,21,51]. The calving-to-weaning period integrates calf viability, maternal care, lactation, calf survival, and maternal nutritional demands.

Greater milk production alone should not be equated with greater biological efficiency because increased lactational output also increases nutrient requirements. Under restricted forage conditions, the relevant phenotype may instead be the capacity to support adequate calf growth while preserving maternal body condition and subsequent reproductive function [21,107].

Likewise, specific behavioral mechanisms should not be attributed to heterosis unless directly measured. Maternal performance is more appropriately considered an integrated phenotype incorporating genetic, physiological, lactational, and behavioral components.

Overall, established nutritional mechanisms and less-certain genotype-associated mechanisms should remain clearly separated. Greater performance of crossbred females under some restricted nutritional environments is most appropriately interpreted as an integrated expression of heterosis, maternal performance, and nutritional robustness rather than evidence for a single physiological mechanism.

### 5.3. Practical Management Implications

The evidence supports two complementary management strategies: structured crossbreeding and nutritional management appropriate to the production environment [10,28,49]. Crossbreeding can improve reproductive and maternal performance through heterosis and breed complementarity, whereas adequate forage availability and nutritional management allow expression of genetic potential.

Management options include adjusting stocking rate, improving pasture allocation and monitoring, strategically supplementing during periods of nutritional deficit, and selecting crossbreeding systems appropriate to available forage resources and climatic conditions [49,110,111]. Importantly, the objective is not simply to maximize nutritional inputs or biological output, but to align cow genotype and nutrient requirements with the production environment [112].

This distinction has practical implications for genotype selection. A genotype that performs well under high-input conditions may not necessarily maximize cumulative reproductive efficiency where forage availability is seasonally restricted. Conversely, crossbred females showing greater robustness under nutritional constraint may be particularly valuable in variable grazing environments. However, direct evidence remains insufficient to prescribe a single crossbreeding strategy across production systems.

### 5.4. Climate Variability and Broader Genotype × Environment Interactions

Climate variability can affect reproduction through both nutritionally mediated and direct physiological pathways. Drought and altered precipitation can reduce forage biomass and quality, producing a pathway described as follows:

Climate variability → forage availability/quality → nutrient intake → energy balance/BCS → metabolic–endocrine function → reproduction.

Heat load can additionally affect feed intake, thermoregulation, endocrine and ovarian function, oocyte and embryo competence, and pregnancy establishment independently of forage availability. Consequently, reproductive decline during hot or drought-prone periods should not automatically be attributed to nutrition alone.

Genetic background may modify both pathways. Breed types differ in heat tolerance, mature size, milk potential, grazing characteristics, and environmental adaptation. However, superior performance of a genotype under combined heat and forage restriction represents broader genotype × environment adaptation unless a differential response specifically to nutritional exposure can be demonstrated.

Future studies should therefore distinguish nutritional G × E, reflecting differential responses to HA or nutrient availability, from broader climatic G × E, in which temperature, drought, and other environmental stressors may act directly or indirectly through nutrition.

### 5.5. Biological-to-Economic Pathways

The economic value of reproductive improvement arises from converting a greater proportion of breeding females into saleable calf output. The biological-to-economic pathway can be represented as follows:

Pregnancy → calving → calf survival/weaning → calves weaned per cow exposed → kg calf weaned per cow exposed → gross calf revenue.

Accordingly:

Gross calf revenue per cow exposed = kg calf weaned per cow exposed × calf value per kg

and:

kg calf weaned per cow exposed = probability of weaning a calf × mean calf weaning weight.

These measures connect reproductive biology more directly with economic performance than pregnancy rate alone. Crossbreeding may influence this pathway through fertility, maternal performance, calf survival, weaning weight, cow longevity, and replacement requirements [20,21,51,87]. However, greater calf output does not necessarily mean greater profitability if it is accompanied by increased mature cow size, feed requirements, breeding costs, or management complexity.

Similarly, improved nutritional management may increase BCS, reproductive success, and calf output but can require changes in stocking rate, supplementation, land use, forage management, infrastructure, or labor. The relevant economic endpoint is therefore:

Net return per cow exposed = calf-output value − cow maintenance − replacement − nutritional/forage-management − breeding-system costs.

Cow longevity is particularly important because reproductive failure increases replacement requirements. If maternal heterosis improves productive longevity, economic benefits may extend beyond annual calf output [30,51,58,113]. Quantification requires longitudinal analyses incorporating replacement costs, salvage value, lifetime productivity, and age structure.

Contextual evidence from tropical cattle should be interpreted separately from direct reproductive G × N experiments. Bunning et al. (2019) [105] synthesized 518 heterosis estimates from 42 studies and found that heterosis differed by trait class and breed combination, whereas the climate grouping was not a significant modifier in their meta-analysis. These estimates do not establish a controlled genotype × nutritional-environment effect on beef cow–calf reproduction and are therefore classified as Level IV supportive evidence in Data File S1.

Formal economic evaluations of G × N remain limited. Consequently, large biological differences reported in individual studies, including model-derived differences in probability of weaning a calf per cow served [48], should not be translated directly into universal monetary benefits. Partial-budget and whole-farm analyses incorporating genotype, HA, reproductive losses, calf weight, longevity, replacement rate, feed costs, and price variability are needed.

### 5.6. Reproductive Efficiency and Environmental Sustainability

Improved reproductive efficiency may reduce the resource and GHG burden per unit of saleable calf output because cows that fail to conceive, maintain pregnancy, or wean a calf continue to consume resources without producing equivalent saleable output during that cycle.

A useful biological-efficiency metric is as follows:

For an annual exposed-cow denominator, kilograms of calf weaned per cow exposed can be expressed as the observed weaning proportion multiplied by mean calf weaning weight; this is an accounting identity, not an estimated G × N effect.

Environmental performance can similarly be expressed in terms of emissions intensity:

$$\mathrm{GHG}\;\mathrm{emissions}\;\mathrm{intensity}\;=\;\frac{\mathrm{Total}\mathrm{GHG}\mathrm{emissions}\mathrm{from}\mathrm{the}\mathrm{production}\mathrm{system}\;}{\mathrm{kg}\mathrm{of}\mathrm{saleable}\mathrm{calf}\mathrm{output}}$$

In principle, improvements in pregnancy maintenance, calf survival, weaning rate, or calf growth may increase saleable output relative to cow-maintenance requirements and thereby reduce emissions intensity under otherwise comparable conditions. Crossbreeding and nutritional management could contribute indirectly through their effects on reproductive and maternal performance; these pathways should not be presented as measured environmental benefits of reproductive G × N in the reviewed studies.

However, reduced emissions intensity does not necessarily mean reduced absolute emissions. Improvements in reproduction may coincide with changes in herd size, stocking rate, cow size, supplementation, feed intake, or total production. Similarly, reproductive efficiency alone cannot be used to infer effects on land use, water use, pasture condition, or biodiversity.

These relationships provide a biological rationale for hypothesizing lower greenhouse-gas emissions intensity per unit of saleable calf output when reproductive efficiency improves, but the reviewed reproductive G × N studies did not directly quantify greenhouse-gas emissions, resource use, or life-cycle impacts. Accordingly, environmental benefits remain untested hypotheses requiring whole-system or life-cycle assessments that account for herd structure, replacement rate, feed resources, stocking density, land use, and total production.

### 5.7. Strengths, Limitations, and Interpretation of the Evidence

The evidence synthesized in this review supports important independent effects of genotype and nutritional environment on reproductive performance, but several limitations constrain inference regarding their interaction.

First, substantial heterogeneity exists among studies in breed composition, crossbreeding structure, parity, physiological stage, grazing management, supplementation, and duration of nutritional exposure. Consequently, purebred and crossbred represent biologically diverse populations, and responses observed for one breed combination should not be generalized to others.

Second, HA and nutritional environment were not consistently defined or quantified. Studies measured forage availability, quality, stocking pressure, supplementation, and animal nutritional status differently. Moreover, HA represents environmental exposure rather than realized nutrient intake, and relatively few studies simultaneously measured HA, intake, energy balance, and longitudinal BCS.

Third, reproductive endpoints differed substantially, including ovarian activity, conception, pregnancy, pregnancy loss, calving, calf survival, weaning, calving interval, and cumulative reproductive outcomes. These endpoints represent different biological stages and are not interchangeable.

Fourth, selected pasture-based dairy studies were included as comparative evidence. Dairy systems differ from beef cow–calf systems in genetic selection, milk production, nutritional demand, management, and reproductive endpoints. Dairy evidence therefore supports broader biological interpretation but should not be used to estimate beef cow–calf effects directly.

Fifth, production environments differed in forage resources, rainfall, stocking pressure, supplementation, heat load, breed composition, and management. Nutritional and climatic exposures also frequently covary, limiting attribution of observed responses specifically to nutrition.

Sixth, inferential strength varied substantially. The synthesis included controlled experiments, long-term grazing studies, observational studies, mechanistic evidence, and modeling analyses, but only a small proportion directly evaluated genotype under contrasting nutritional environments. Independent genotype and nutritional effects therefore should not be interpreted as G × N without evidence of differential genotype responses.

Seventh, heterogeneity in study design, genotype definition, nutritional exposure, endpoints, and statistical analysis precluded meaningful quantitative pooling. Numerical effects from individual studies, including differences in calves weaned per cow exposed, should therefore be interpreted within their specific experimental and production contexts.

Finally, G × N was not evaluated using standardized approaches, and the evidence base remains geographically and genetically uneven. Publication and reporting bias are also possible, particularly because favorable heterosis or nutritional responses may receive greater emphasis, and publication bias was not formally quantified. In addition, the review was not prospectively registered, duplicate independent screening and formal study-level risk-of-bias assessment were not performed, and the aggregate search counts were generated from multiple complementary searches that may overlap within and across databases. These features limit direct application of the complete PRISMA 2020 study-selection framework and should be considered when interpreting the comprehensiveness of the evidence set.

Collectively, these limitations mean that the synthesis is strongest in identifying biologically consistent patterns, context-dependent responses, and evidence gaps, rather than providing a universal quantitative estimate of crossbreeding, nutritional, or G × N effects. Future studies should use standardized nutritional exposures and explicit G × N models; repeated measures of intake, BCS, metabolic status, and reproductive outcomes; and common cumulative endpoints such as calves and kilograms of calf weaned per cow exposed.

The principal need for future research is not simply additional studies of genotype or nutrition independently. Instead, hypothesis-driven experiments should determine when, where, and through which biological pathways the nutritional environment modifies genotype-associated reproductive performance. The present synthesis generates five testable hypotheses.

**H1.**  *Crossbred females exhibit a smaller decline in calves weaned per cow exposed as herbage allowance decreases. As conceptualized in Figure 3, this hypothesis defines nutritional robustness as a smaller decline in reproductive or cow–calf performance as nutritional conditions become restrictive, rather than as superior average performance across environments. Testing this hypothesis requires factorial or longitudinal comparisons of defined genotypes across contrasting HA levels. The primary endpoint should be calves weaned per cow exposed, with losses partitioned across breeding-to-pregnancy, pregnancy-to-calving, and calving-to-weaning*.

**H2.**  *Greater nutritional robustness of some crossbred females is mediated partly through preservation or recovery of body condition rather than universally lower maintenance energy requirements. Future studies should combine longitudinal BCS and body-reserve measurements with intake, energy balance, milk production, and metabolic indicators such as insulin, IGF-1, and leptin. Mediation or structural-equation approaches could then test whether BCS trajectory links nutritional exposure with reproductive performance*.

**H3.**  *G × N may be more strongly expressed during calving-to-weaning than during pregnancy establishment. Studies should therefore avoid pregnancy rate as the sole reproductive endpoint and quantify conception or early pregnancy success, pregnancy loss, calving success, calf survival, and weaning success separately. Stage-specific models would permit direct comparison of interaction magnitude across the reproductive continuum*.

**H4.**  *Maternal heterosis may contribute more strongly to lifetime kilograms of calf weaned than is apparent from annual pregnancy rate alone. Long-term cohorts should quantify lifetime calves and kilograms of calf weaned per female entering the breeding herd, including females that produce no calves, while distinguishing direct heterosis, maternal heterosis, breed additive effects, and breed complementarity*.

**H5.**  *Nutritional robustness differs among breed combinations and crossbreeding systems and is not a generic property of crossbred cattle. Multi-breed, multi-environment studies are needed to determine which breed combinations maintain reproductive performance most effectively under defined nutritional constraints and whether advantages observed in F1 females persist in rotational or later-generation populations*.

Testing these hypotheses will require more precise characterization of environmental exposure than is typical of much of the existing literature. Future studies should quantify HA, forage mass and quality, stocking pressure, supplementation, animal nutritional status, longitudinal BCS, rainfall, and THI or other heat-load measures. Designs that evaluate genotype under combinations of adequate versus restricted nutrition and contrasting heat load would help distinguish nutritional G × E from broader climatic G × E and identify potential genotype × nutrition × climate interactions.

Genomic information should also be integrated with detailed reproductive and physiological phenotypes. Genome-wide association, genomic prediction, and reaction-norm or genomic G × E approaches may help identify genetic variation associated with maintenance of reproductive performance across nutritional environments. These approaches will be most informative when environmental exposure is measured quantitatively, and reproductive phenotypes are consistently defined. Hierarchical and Bayesian models are particularly suitable for longitudinal, multi-stage datasets because they can accommodate repeated observations, farm and year effects, heterogeneous exposures, and uncertainty in stage-specific transitions.

Biological responses should also be linked to economic and environmental outcomes. Partial-budget and whole-system analyses are needed to determine whether G × N effects on pregnancy, calf survival, weaning weight, cow longevity, and replacement rate generate positive net returns after accounting for supplementation, forage management, land, breeding-system, and replacement costs. Likewise, life-cycle or whole-system assessments should evaluate whether gains in calves or kilograms of calf weaned per cow exposed reduce GHG emissions intensity after accounting for differences in feed requirements, mature cow size, stocking, and other resource inputs.

Collectively, these priorities shift the research agenda from asking whether crossbreeding or improved nutrition independently enhance reproductive performance toward determining which genotypes are most robust, under which nutritional environments, at which reproductive stages, and through which physiological mechanisms. Table 3 summarizes the principal evidence gaps, and study designs needed to address these questions.

Table 3 summarizes the principal evidence gaps and identifies testable hypotheses and research approaches needed to advance understanding of reproductive genotype × nutritional environment interactions in grazing cattle. However, improved experimental design alone will not ensure comparability across studies. Consistent reporting of genotype, nutritional exposure, physiological state, environmental conditions, reproductive outcomes, and statistical interaction estimates is also essential. Accordingly, Table 4 proposes a minimum reporting framework to improve methodological transparency, facilitate cross-study comparisons, and support future quantitative evidence synthesis.

Together, the hypothesis-driven priorities summarized in Table 3 and the minimum reporting framework proposed in Table 4 provide a basis for determining when, where, and through which biological pathways nutritional environment modifies genotype-associated reproductive performance. The evidence inventory also indicates that future quantitative synthesis is constrained not only by biological heterogeneity but by inconsistent reporting of denominators, genotype × nutrition cell sizes, stage-specific reproductive outcomes, interaction estimates, and overlapping cohorts. More consistent study design and reporting should help distinguish true biological heterogeneity from differences arising from phenotype definition, environmental characterization, management, or analytical approach and provide a stronger foundation for future quantitative synthesis and genotype-specific nutritional recommendations for grazing cow–calf systems.

## 6. Conclusions

Reproductive efficiency in grazing beef cow–calf systems reflects the combined influences of genotype, nutritional environment, maternal function, and reproductive losses occurring from breeding through weaning. The available literature suggests that crossbreeding is frequently associated with favorable reproductive and maternal outcomes and that nutritional conditions are related to body condition, reproductive function, calf survival, and cumulative productivity. However, the magnitude and consistency of these relationships vary across breeds, nutritional exposures, physiological stages, and production systems. Direct evidence of reproductive genotype × nutritional environment (G × N) interactions remains limited. The two publications classified as direct reproductive G × N evidence originated from a shared Uruguayan grazing research program; one reported a stage-specific interaction, particularly during the calving-to-weaning period, whereas the other included an interaction term without separately reporting its estimate. Consequently, these findings do not constitute independent experimental replication or establish nutritional robustness as a universal characteristic of crossbred cattle. Furthermore, heterogeneity in study designs and outcomes, the contribution of observational evidence, the absence of formal risk-of-bias assessment, and the lack of quantitative pooling limit causal interpretation and generalizability. Evaluating calves and kilograms of calf weaned per cow exposed may provide a more comprehensive assessment of reproductive efficiency than pregnancy rate alone. Independent, longitudinal factorial studies with clearly defined genotypes, quantified nutritional exposures, standardized reproductive outcomes, and explicitly reported interaction estimates are needed to determine the reproducibility and practical significance of reproductive G × N interactions across diverse grazing beef production systems. The 36 classified records represent a purposive narrative evidence inventory that was expanded during revision; they should not be interpreted as a deduplicated, consecutively screened database sample. Numerical estimates from primary articles that remain unverified should be considered provisional.

## Figures and Tables

**Table 1:** Definitions and interpretation of reproductive and cumulative production outcomes used in the evidence synthesis.

Outcome	Recommended Definition	Conceptual Stage
**Pregnancy rate**	Proportion of eligible/exposed females diagnosed pregnant at a defined time point	Breeding → pregnancy
**Conception rate**	Proportion of breeding/insemination events resulting in an established pregnancy, with denominator explicitly defined	Fertilization/pregnancy establishment
**Pregnancy loss**	Proportion of established pregnancies subsequently lost between specified diagnostic or reproductive time points	Pregnancy → calving
**Calving rate**	Proportion of females exposed to breeding that subsequently calve; denominator should be stated explicitly	Breeding → calving
**Calf survival**	Proportion of live-born calves surviving to a specified age or weaning; time interval should be stated	Calving → weaning
**Weaning rate (or)** **Calves weaned/cow exposed**	Proportion of cows exposed to breeding that ultimately wean a calf, unless another denominator is explicitly stated	Entire breeding → weaning cascade
**kg calf weaned/cow exposed**	Total calf weight at weaning divided by number of females originally exposed to breeding	Cumulative biological/productive efficiency
**Calving interval**	Number of days between successive calvings	Longitudinal reproductive timing
**Days to conception**	Number of days from a defined biological or management starting point (e.g., calving or onset of the breeding period) to conception	Reproductive timing

**Table 2:** Production-environment contexts relevant to interpretation of genotype × nutritional environment relationships.

Production Environment	Nutritional Challenge	Genetic/Reproductive Context	Key Interpretive Issue
Temperate/native grassland	Seasonal HA and forage variation; stocking pressure	Beef purebreds and crosses; seasonal cow–calf	Strong relevance to HA × genotype
Semi-arid rangeland	Rainfall and forage variability	Beef breeds/crosses; extensive cow–calf	Nutrition overlaps broader environmental stress
Subtropical grazing	Seasonal forage quantity/quality; heat load	*Bos taurus* and *Bos indicus*-influenced genetics	Breed complementarity and climatic adaptation complicate G × N
Seasonal pasture dairy	Pasture seasonality; high lactational demand	Dairy purebreds/crosses; compact breeding	Comparative evidence; endpoints differ from beef

The apparent consistency of crossbreeding advantages across regions should not be interpreted as a geographically invariant response. Rather, expression of heterosis and G × N depends on the nutritional, climatic, genetic, reproductive, and management context.

**Table 3:** Principal evidence gaps and priorities for future research on genotype × nutritional environment effects on reproductive efficiency in grazing cattle.

Evidence Gap	Testable Hypothesis/Question	Why It Matters	Recommended Approach
Few direct G × HA reproductive experiments	**H1:** Does the decline in calves weaned per cow exposed with decreasing HA differ between crossbred and purebred females?	Directly tests genotype-specific nutritional robustness	Multi-site factorial genotype × HA experiments with stage-specific reproductive outcomes
Limited longitudinal BCS/metabolic data	**H2:** Do crossbred and purebred females differ in their ability to maintain BCS during nutritional restriction (robustness) and to regain BCS following nutritional restoration (resilience), and are these responses associated with subsequent reproductive performance?	Distinguishes maintenance of physiological function during nutritional challenge (robustness) from recovery following challenge (resilience) and identifies potential pathways linking nutritional responses to reproductive performance.	Longitudinal genotype × nutritional-treatment studies with repeated BCS, intake, milk production, metabolic indicators, and reproductive measurements during restriction and recovery.
Limited stage-specific G × N evidence	**H3:** Is G × N stronger during calving-to-weaning than during pregnancy establishment?	Identifies where along the reproductive continuum G × N is expressed	Longitudinal stage-transition analysis
Sparse lifetime productivity data	**H4:** Does maternal heterosis contribute more strongly to lifetime kilograms of calf weaned per female entering the breeding herd than to annual pregnancy rate?	Annual fertility may underestimate cumulative maternal heterosis benefits	Multi-year lifetime cow cohorts with cumulative calf-output measures
Breed-specific evidence and limited generalizability	**H5:** Does nutritional robustness differ among breed combinations and crossbreeding systems rather than representing a general property of crossbred cattle?	Prevents treating crossbreeding as a uniform biological effect	Multi-breed × multi-environment comparisons
Limited genomic information	Which genomic features predict reaction norms for reproductive performance across nutritional environments?	Biological and genetic bases of robustness remain unclear	Genomic reaction-norm and G×E models linked to longitudinal phenotypes
Few economic analyses	Under what feed- and calf-price conditions does reproductive G × N improve net return?	Determines economic relevance and adoption potential	Partial-budget and whole-system modeling with sensitivity analysis
Limited environmental-footprint integration	Does improved cumulative reproductive efficiency reduce GHG emissions intensity?	Tests whether reproductive gains translate into environmental benefits	LCA integrated with reproductive and production-system models

**Table 4:** Proposed minimum reporting framework for studies evaluating genotype × nutritional environment effects on reproductive performance in grazing cattle.

Domain	Minimum Information to Report	Rationale
**Genotype and crossbreeding**	Breed(s); breed fractions; sire and dam breed; crossbreeding system; generation; expected or estimated heterozygosity/retained heterosis, when applicable	Distinguishes breed additive effects, heterosis, and breed complementarity and permits comparison among crossbreeding systems
**Nutritional environment**	Operational definition of HA and units; forage mass/biomass; forage quality; stocking rate or stocking pressure; supplementation type, amount, and timing; duration and physiological timing of nutritional treatment	Defines the nutritional contrast and allows the magnitude and timing of nutritional challenge to be compared among studies
**Animal physiological state**	Parity; age; BW; BCS at relevant baseline(s); longitudinal BCS or BCS change; lactation status; milk yield or milk potential when available; postpartum interval	Characterizes animal-level differences that may modify nutritional requirements and reproductive responses
**Physical environment**	Location and production system; rainfall/precipitation; temperature and THI or other heat-load metric; drought index or other relevant climatic indicators; season/year	Separates nutritional exposure from broader climatic and temporal environmental effects
**Reproductive outcomes**	Number of females exposed to breeding; breeding method and season; timing and method of pregnancy diagnosis; denominator for each outcome; pregnancy success; pregnancy loss; calving success; calf survival; weaning success	Allows reproductive attrition to be partitioned across breeding → pregnancy → calving → weaning rather than relying on pregnancy rate alone
**Cumulative cow–calf output**	Calves weaned per cow exposed; kilograms of calf weaned per cow exposed; weaning age and whether weights are actual or age-adjusted	Provides integrated outcomes incorporating reproductive success, calf survival, and preweaning performance
**G × N statistical analysis**	Explicit genotype, nutrition, and genotype × nutrition terms; sample size by genotype × nutrition combination; genotype-specific outcome estimates within each nutritional condition; the estimated interaction contrast (difference-in-differences or equivalent model-based interaction parameter) with 95% confidence or credible interval; interaction *p*-value or posterior probability, as appropriate; and repeated-measures/random-effect structure where applicable	Distinguishes genuine genotype × nutrition interaction from independent main effects or genotype differences within individual nutritional conditions; quantifies the direction, magnitude, and precision of interaction effects; and facilitates comparison and quantitative synthesis across studies.
**Longitudinal and carry-over exposure**	Previous nutritional treatment/exposure; previous BCS or body-reserve trajectory; duration of restriction and recovery; timing of refeeding; previous reproductive/lactational state when relevant	Permits evaluation of nutritional memory, recovery, and carry-over effects across reproductive stages or production cycles
**Management context**	Grazing management; breeding management; temporary weaning or calf management; health interventions; pasture allocation strategy and other major management differences among treatments	Identifies management practices that may modify or confound apparent genotype × nutrition responses

## Data Availability

No new data were created or analyzed in this study. Data sharing is not applicable to this article.

## Supplementary Materials

The following supporting information can be downloaded at: https://www.mdpi.com/article/10.3390/ani16213344/s1, Supplementary Table S1 reports available database search combinations and aggregate retrieval counts (19,604 database query returns and 253 citation-search returns; 19,857 combined, not unique records). Supplementary Figure S1 is an evidence-identification transparency diagram for this structured narrative review, not a PRISMA 2020 flow diagram. Data File S1 contains the publication-level evidence inventory (Level I: 2; II: 14; III: 9; IV: 11), source-verification notes, cohort linkage, and audit status. Historical deduplication, screening, eligibility and exclusion counts are unavailable. Supplementary Figure S1 caption: Evidence-identification transparency diagram for a structured narrative review. Database and citation-search returns are aggregate, overlapping query returns, not deduplicated records. The 36 included records constitute a purposively assembled discussed-publication inventory. Historical deduplication, screening, eligibility, and exclusion counts were not retained; this figure is not a PRISMA 2020 flow diagram.
