# Supplementary material for: Genotype × Nutritional Environment Interactions in Grazing Beef Cow-Calf Systems: Evidence for Crossbreeding and Reproductive Efficiency from Breeding to Weaning
Source: Animals (Basel). 2026 Oct 23;16(21):3344. doi: 10.3390/ani16213344 (PMC13761450; doi:10.3390/ani16213344)
Supplement: Supplementary file 1 [file animals-16-03344-s001.zip › Supplementary Table S1_R3.pdf]

Supplementary Table S1. Database-specific literature-search strategies and retrieval counts used for the structured narrative evidence synthesis (for PRISMA-S-informed reporting).

Database/source	Coverage/date searched	Search combinations / strategy reported	Aggregate records retrieved*
**PubMed**	Inception–Through September 16 2026	(1) cattle AND crossbreeding AND fertility; (2) heterosis AND reproductive performance; (3) herbage allowance AND pregnancy; (4) stocking rate AND reproduction; (5) genotype AND nutrition AND fertility; (6) grazing AND reproductive efficiency;<br>plus<br>cattle AND genotype by environment; cattle AND genotype by nutrition; cattle AND genotype by nutrition by environment	2,824
**Web of Science Core Collection**	Inception–Through September 16 2026	Same six search combinations: (1) cattle AND crossbreeding AND fertility; (2) heterosis AND reproductive performance; (3) herbage allowance AND pregnancy; (4) stocking rate AND reproduction; (5) genotype AND nutrition AND fertility; (6) grazing AND reproductive efficiency;<br>plus<br>cattle AND genotype by environment; cattle AND genotype by nutrition; cattle AND genotype by nutrition by environment	7,609
**Scopus**	Inception–Through September 16 2026	Same six search combinations: (1) cattle AND crossbreeding AND fertility; (2) heterosis AND reproductive performance; (3) herbage allowance AND pregnancy; (4) stocking rate AND reproduction; (5) genotype AND nutrition AND fertility; (6) grazing AND reproductive efficiency;<br>plus<br>cattle AND genotype by environment; cattle AND genotype by nutrition; cattle AND genotype by nutrition by environment	6,282
**CAB Abstracts**	Inception–Through September 16 2026	Same six search combinations: (1) cattle AND crossbreeding AND fertility; (2) heterosis AND reproductive performance; (3) herbage allowance AND pregnancy; (4) stocking rate AND reproduction; (5) genotype AND nutrition AND fertility; (6) grazing AND reproductive efficiency;<br>plus<br>cattle AND genotype by environment; cattle AND genotype by nutrition; cattle AND genotype by nutrition by environment	2,889

Database/source	Coverage/date searched	Search combinations / strategy reported	Aggregate records retrieved*
**Citation searching**	Through September 16 2026	Reference lists of eligible primary studies and relevant review articles	253

* Records retrieved are aggregate numbers returned across separately run complementary search combinations and interaction-specific searches before screening and deduplication. These totals may contain within-database and between-database overlap and therefore are not unique PRISMA identification counts. They should not be used to infer the number of records screened or excluded. Data File S1 contains the final 36 publication-level records used as the defined classified evidence inventory for the manuscript, comprising Level I (n = 2), Level II (n = 14), Level III (n = 9), and Level IV (n = 11) records, with evidence role, cohort linkage, study characteristics, outcomes, and source information.

Reporting note. This table summarizes the reported database-search strategies and aggregate retrieval counts for this structured narrative review. The combined 19,857 query returns (19,604 database returns and 253 citation-search returns) represent overlapping search results rather than deduplicated unique records. The final evidence inventory comprises 36 publication-level records (Level I, n = 2; Level II, n = 14; Level III, n = 9; Level IV, n = 11). Related publications originating from shared experimental cohorts are linked and should not be interpreted as independent studies. Historical records of deduplication, title/abstract screening, full-text eligibility assessment, and exclusions with reasons were not retained; consequently, these counts cannot be reconstructed reliably. The review was not prospectively registered and did not employ duplicate independent screening or formal study-level risk-of-bias assessment. Supplementary Figure S1 is therefore presented as an evidence-identification transparency diagram for this structured narrative review, not as a fully compliant PRISMA 2020 flow diagram. The supplementary search reporting is informed by relevant PRISMA-S principles but does not establish complete PRISMA-S or PRISMA 2020 compliance.
